# Astrocytic Neuroligin-3 influences gene expression and social behavior, but is dispensable for synapse number

**DOI:** 10.1038/s41380-024-02659-6

**Published:** 2024-07-13

**Authors:** Liming Qin, Zhili Liu, Sile Guo, Ying Han, Xiankun Wang, Wen Ren, Jiewen Chen, Hefu Zhen, Chao Nie, Ke-Ke Xing, Tao Chen, Thomas C. Südhof, Yuzhe Sun, Bo Zhang

**Affiliations:** 1 School of Chemical Biology and Biotechnology, Peking University Shenzhen Graduate School, Shenzhen, 518055 China; 2 Institute of Neurological and Psychiatric Disorders, Shenzhen Bay Laboratory, Shenzhen, 518132 China; 3 BGI Research, Shenzhen, 518083 China; 4 Department of Obstetrics and Gynaecology, The Chinese University of Hong Kong, Hong Kong, China; 5 Department of Anatomy and K.K. Leung Brain Research Centre, Fourth Military Medical University, Xi’an, 710032 China; 6 Department of molecular and cellular physiology, Howard Hughes Medical Institute, Stanford University School of Medicine, Stanford, CA 94043 USA; 7 BGI Research, 102601 Beijing, China; 8 Shenzhen Key Laboratory of Neurogenomics, BGI-Shenzhen, Shenzhen, 518120 China

**Keywords:** Autism spectrum disorders, Neuroscience, Molecular biology

## Abstract

Neuroligin-3 (*Nlgn3*) is an autism-associated cell-adhesion molecule that interacts with neurexins and is robustly expressed in both neurons and astrocytes. Neuronal *Nlgn3* is an essential regulator of synaptic transmission but the function of astrocytic *Nlgn3* is largely unknown. Given the high penetrance of *Nlgn3* mutations in autism and the emerging role of astrocytes in neuropsychiatric disorders, we here asked whether astrocytic *Nlgn3* might shape neural circuit properties in the cerebellum similar to neuronal *Nlgn3*. Imaging of tagged Nlgn3 protein produced by CRISPR/Cas9-mediated genome editing showed that Nlgn3 is enriched in the cell body but not the fine processes of cerebellar astrocytes (Bergmann glia). Astrocyte-specific knockout of *Nlgn3* did not detectably alter the number of synapses, synaptic transmission, or astrocyte morphology in mouse cerebellum. However, spatial transcriptomic analyses revealed a significant shift in gene expression among multiple cerebellar cell types after the deletion of astrocytic *Nlgn3*. Hence, in contrast to neuronal *Nlgn3*, astrocytic *Nlgn3* in the cerebellum is not involved in shaping synapses but may modulate gene expression in specific brain areas.

## Introduction

Neuroligins constitute a family of postsynaptic cell-adhesion molecules (Nlgn1-Nlgn4) that interact with presynaptic neurexins [1]. Different neuroligin mRNAs are co-expressed in the same neurons where they are localized to distinct types of synapses [2–9]. Extensive studies using mice with genetic deletions of neuroligins suggested that neuronal neuroligins play essential roles in shaping the properties, but not the initial formation, of synapses. In cerebellar Purkinje cells, for example, the deletion of *Nlgn1* and *Nlgn3* each decreased climbing-fiber synaptic transmission, with their combined deletion producing additive effects [10]. The deletion of *Nlgn1* and *Nlgn3* in Purkinje cells, however, did not affect the parallel-fiber synaptic transmission, presumably because parallel-fiber synapses utilize Cbln1-GluD2 complexes as postsynaptic receptors for presynaptic neurexins [11, 12]. Conversely, the deletion of *Nlgn2* in the Purkinje cells selectively reduced inhibitory synaptic transmission; this impairment was aggravated by the additional deletion of *Nlgn3* [10]. Moreover, deletion of *Nlgn1*, *Nlgn2*, and *Nlgn3* in cerebellar stellate cells suppressed extrasynaptic NMDA-receptors mediated excitatory synaptic transmission but only modestly impaired inhibitory synaptic transmission onto stellate cells. This manipulation again did not affect AMPA-receptor- (AMPAR-) mediated parallel-fiber synaptic transmission [13], presumably because similar to Purkinje cells, parallel-fiber synapses of stellate cells utilize postsynaptic Cbln1-GluD1 and/or -GluD2 complexes as neurexin ligands [14]. At the subsynaptic level, Nlgn3 regulates AMPAR-mediated synaptic transmission by clustering the postsynaptic AMPARs/PSD95 complex [15]. These results, together with other studies in mice [16–23] and invertebrates [24–26] established the importance of neuronal neuroligins as a central organizer of synaptic transmission.

**Fig. 1:**
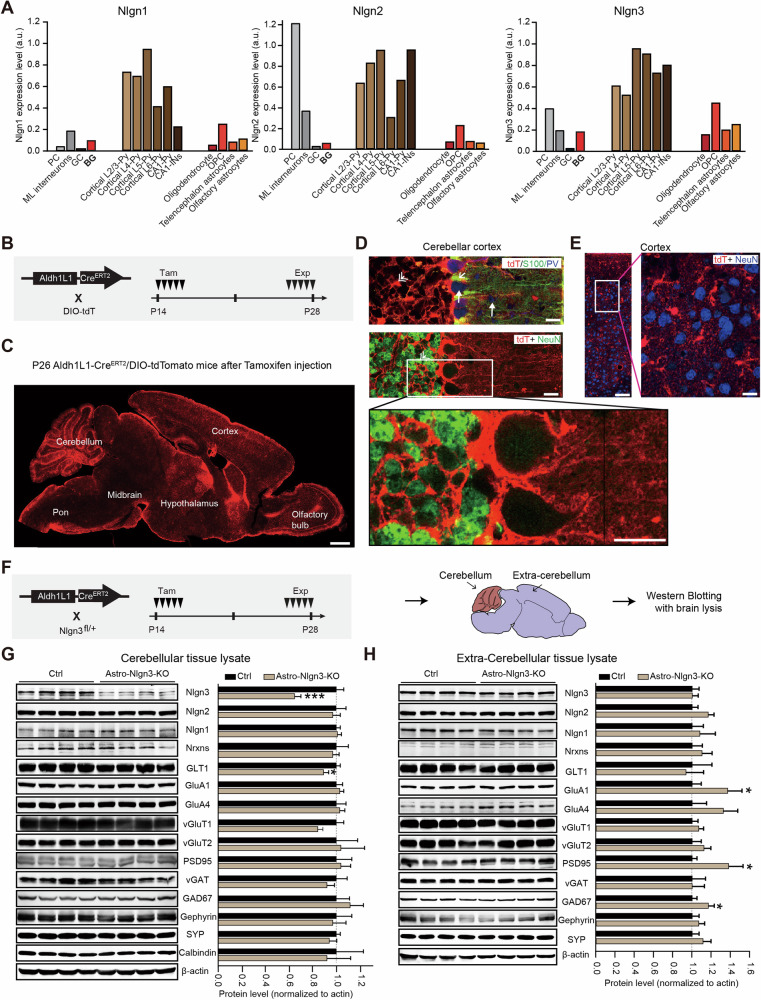
Nlgn3 protein is enriched in cerebellar astrocytes. **A** Nlgn1-Nlgn3 expression profile in different types of neurons and glia using single-cell RNAseq data from Mousebrain.org. **B** Generation of Aldh1L1-Cre/ERT2-DIO-tdTomato transgenic mice. Animals were injected with tamoxifen between P14 and P18 and staining with cell-specific markers was performed between P24 and P28. **C** Representative montage of the sagittal section for Aldh1L1-Cre/ERT2; Ai14 double transgenic mouse injected with tamoxifen. **D** Representative images of cerebellum astrocytes in the Aldh1L1-Cre/ERT2 mouse showing co-staining for S100 protein (green), but not PV (blue) and NeuN (green). Arrows point to Pv-positive PC and interneurons. **E** Representative images of cortex astrocytes in the Aldh1L1-Cre/ERT2 mouse showing Cre-recombination broadly in cortical astrocytes. **F** Generation of Nlgn3^fl/y^/Aldh1L1-CreERT2 transgenic mice. Animals of control and Aldh1L1-Nlgn3-KO were injected with tamoxifen between P14 and P18 and further protein analysis with tissue lysate was performed between P24 and P28. **G**, **H** Representative quantitative immunoblots and summary graphs of protein levels in the cerebellar tissue lysate and extra-cerebellar tissue lysate of P24 Aldh1L1-Nlgn3-KO mice and littermate control after tamoxifen treatment. All data presented as means ± SEM; *p < 0.05; **p < 0.01; ***p < 0.001 by Welch’s *t*-test (*n* = 10 littermate pairs).

In addition to the neuronal neuroligins, single-cell RNAseq data identified neuroligin mRNAs in astrocytes, suggesting a potential role of neuroligins in astrocytes (Fig. 1A, also see [27–29]). The role of astrocytes in organizing synaptic connectivity in the brain has been much debated [30–34]. Moreover, recent evidence has strongly implicated astrocytes in neuropsychiatric disorders [35–38]. In the cerebellum, for example, multiple types of astrocytes are readily identified, including Bergmann glia, velate astrocytes, and astrocytes in the deep cerebellar nuclei. Bergmann glia are typical astrocytes with a large cell body and complex processes extending into the molecular layer of the cerebellar cortex. In Bergmann glia, perisynaptic processes contain high concentrations of both Ca^2+^-permeable AMPA receptors and GLT1 glutamate transporters [39–41]. Overexpressing Ca^2+^-impermeable AMPARs [42], genetically deleting AMPARs [43], or suppressing the glutamate transporter GLAST [44] in Bergmann glia impaired the clearance of synaptic glutamate, thereby altering synaptic transmission onto Purkinje cells. Astrocytes express a large number of transmembrane proteins (e.g. channels, inotropic and metabotropic receptors, and transporters) in their cell body to maintain homeostasis and signal transduction. Therefore, the localization of a specific protein in cerebellar astrocytes might determine the protein function in the neuronal circuit. Thus, it is critical to examine whether neuroligin mRNAs are translated in astrocytes and whether astrocytic neuroligins, if translated, play a role in synaptic transmission and the function of the brain.

Here, we used CRISPR/Cas9-mediated genomic editing, *Nlgn3* conditional knockout (cKO) mice, and spatial transcriptomic analyses to reveal that *Nlgn3* is enriched in cerebellar astrocytes (i.e., Bergmann glia), where we show that it is primarily located in the cell body. Deletion of astrocytic *Nlgn3* modulates gene expression in multiple cell types and alters mouse behavior without detectably affecting the number and function of synapses. Therefore, both neuronal and astrocytic *Nlgn3* contribute to brain function but via different mechanisms.

## Methods

### Animal maintenance

Mice were housed in the animal facility of the Peking University Shenzhen Graduate School (PKUSZ) with a maximum of 5 mice per cage. All mice were maintained under a 12-hour light/dark cycle at a temperature of 22–25 °C and provided with unrestricted access to tap water and standard chow. Female Nlgn3 heterozygous conditional knockout mice (Nlgn3^fl/+^, JAX#023398) were crossed with male Aldh1L1-CreERT2 (JAX#029655) or Glast-CreER transgenic mice (JAX#012586) to obtain male Nlgn3-cKO mice with the Aldh1L1-CreERT2 or GLAST-CreER transgene on a hybrid C57BL/6/129Sv/CD1 background. Please note that mouse behaviors are known to be strongly influenced by genetic background. Cas9^fl/fl^ mice are obtained from Jackson lab (JAX#024857) and Ai14 mice are obtained from Jackson lab (JAX#007908). C57BL6/J mice were purchased from Guangdong Medical Laboratory Animal Center (China). Male mice at the age of P24-P28 from Nlgn3-cKO (Nlgn3^fl/Y^; Aldh1L1-Cre/ERT2) and littermate control mice (Nlgn3^+/Y^; Aldh1L1-Cre/ERT2) (after tamoxifen injection starting at P14 for 5 days) were used for the experiments. Male mice at age 2-3 months were used for the behavior tests. All animal procedures followed the guidelines approved by the Peking University Shenzhen Graduate School Animal Care and Use Committee and Shenzhen Bay Laboratory Animal Care and Use Committee.

### Tamoxifen injection

See Supplementary Methods for details.

### DNA constructs/plasmids

See Supplementary Methods for details.

### AAV design and production

See Supplementary Methods for details.

### Virus injection surgery

See Supplementary Methods for details.

### Primary cell cultures and transfection

See Supplementary Methods for details.

### Immunoblotting

Immunoblotting was performed with fluorescently labeled secondary antibodies and Licor detection as described previously [10, 13]. See Supplementary Methods for details.

### qRT-PCR

See Supplementary Methods for details.

### Immunofluorescence analysis

See Supplementary Methods for details.

### Immunohistochemistry

Immunohistochemistry experiments and other morphological studies were performed essentially as described previously [10, 13]. See Supplementary Methods for details.

### Electron microscopy

See Supplementary Methods for details.

### Electrophysiology

Electrophysiological experiments were carried out as described previously [10, 13]. See Supplementary Methods for details.

### Spatial transcriptome library preparation

Snap-frozen brain samples were embedded by the OCT (Sakura, USA, 4583#). Sections of 10 µm were obtained from each sample and then used 1 cm^2^ stereo-seq capture chips (MGI, China) to construct a spatial transcriptomics library. The capture spot diameter of the chip is 220 nm, with a center-to-center distance of 500 nm. Sections were incubated at 37 °C for 3 min immediately after being attached to chips rapidly and then fixed in the precooled methanol (Sigma, USA, 34860) at −20 °C for 40 min. The fixed sections were stained by Qubit ssDNA reagent (Thermo Fisher, USA, Q10212), and the integrity of the samples was checked under the fluorescence microscope (Motic, China, P7000). Next, the sections were permeabilized via 37 °C preheated 0.1% pepsin (MGI, China, P7000) in 0.01 mol/L HCl, and incubated at 37 °C for 10 min. After washing out the enzyme, reverse transcription was performed at 42 °C for 2 h and then digested with tissue removal buffer (MGI, China) at 42 °C for 30 min to remove residual tissue. The chips containing cDNA were treated with cDNA-release enzyme (MGI, China) at 55 °C overnight. The released cDNA was amplified and purified, and finally, the DNA nanoball library was generated for sequencing in the single-end 50 + 100 bp strategy on the MGI DNBSEQsequencer (MGI, China).

### Spatial transcriptomic data processing

The raw data of Stereo-seq were preliminarily processed through the Stereomics pipeline and could be visualized from the STOmicswebsite (http://stereomap.cngb.org/). According to the results of ssDNA staining, the cerebellum regions were extracted via in situ data from the capture chips. The bin 50 data from 50 × 50 DNB bins with a diameter of 25 μm were processed using Seurat v4, and normalized by the LogNormalize method. The bin 50 spots with unique molecular identifiers (UMIs) < 200 and mitochondrial genes >5% were removed. Dimension reduction was performed by principal components analysis (PCA), and the number of principal components (PCs) used for generating tSNE plots was determined by the Elbowplot function. Marker genes and differential expressed genes (DEGs) according to the Wilcoxon rank sum test were obtained through FindAllMarkers and FindMarkers functions, respectively. The spatial distribution of marker genes with a threshold of 0.25 log2 fold change and adjusted *p*-value < 0.05 was consistent with the experimental results. The case-control DEGs of each cell type with a threshold of |0.25| log2 fold change and adjusted *p*-value were performed functional enrichment analysis via cluster Profiler v4. The data have been uploaded into the STOmicsDB with accession number STT0000040.

### Pathway activity analysis

The MAYA [45], an R package for quantitative analysis of pathway activity, was used to measure the pathways associated with calcium ions and organelle localization. The ontology gene sets of the mouse from the Molecular Signatures Database (MSigDB) were downloaded and 323 molecular functions and biological processes associated with calcium ions and organelle localization were chosen. The activity of each pathway in each cell was calculated by MAYA, and then the pathway differences were analyzed in each cell type. The thresholds of differential pathways were |log2 fold change|>0.25 and adjusted *p*-value < 0.05.

### Behavioral tests

Behavioral tests were conducted during the day (light-on period) using age-matched male littermates (2-3 months). All mice were handled by the operator for 2–3 min per day for 5 consecutive days for habituation. Rest for at least 1 day between different tests. See Supplementary Methods for details.

### Quantification and statistical analysis

Statistical results were presented as mean ± SEM. Investigators were blinded to animal genotypes. Inter-group comparisons were performed using Welch’s *t*-test. For multiple comparisons, data were analyzed using two-way ANOVA; Cumulative distributions were analyzed using Kolmogorov–Smirnov tests. For behavior tests, two-way ANOVA for open field and the rotarod test, and the two-way ANOVA followed by post hoc comparisons using the Holm–Sidak test for the three-chamber test. Significance levels were set as *p < 0.05; **p < 0.01; ***p < 0.001. Data shown are means ± SEM.

## Results

### *Nlgn3* is robustly expressed in cerebellar astrocytes

To estimate the protein expression levels of Nlgn3 in astrocytes, we used the Aldh1L1-Cre/ERT2 mouse line [46] that enables us to specifically delete astrocytic *Nlgn3*. We validated the astrocyte specificity of this cre line by injecting tamoxifen into mice carrying Aldh1L1-Cre and Cre-dependent tdTomato indicator (Ai14) alleles. We found that Cre-mediated recombination was selectively and broadly present in cerebellar and cortical astrocytes but not in neurons (Fig. 1B–E). Thus, the Aldh1L1-Cre/ERT2 line exhibits high specificity for astrocytes.

After validating the specificity of the Cre line, we crossed male Aldh1L1-Cre/ERT2 mice with female *Nlgn3*^*fl*/+^ mice to obtain male offspring of astrocyte-specific *Nlgn3* KO mice (Nlgn3^fl/Y^; Aldh1L1-Cre/ERT2, referred to as Astro-Nlgn3-KO) and littermate male control mice (Nlgn3^+/Y^; Aldh1L1-Cre/ERT2). Only male mice were examined because this was an economically feasible approach to study littermates that carried the Aldh1L1-Cre/ERT2 allele but differed in the *Nlgn3*^*fl*^ allele since *Nlgn3* is an X-chromosomal gene. Using quantitative immunoblotting, we found that the deletion of *Nlgn3* in astrocytes caused a ~40% loss of Nlgn3 protein in the cerebellum. We only observed a mild reduction in GLT1 levels in the cerebellar homogenate. No other synaptic proteins tested exhibited significant changes in levels; in particular, vGluT1 (a presynaptic marker of parallel-fiber synapses) and vGluT2 (a presynaptic marker of climbing-fiber synapses) were not altered (Fig. 1F, G). Notably, we did not observe a significant decrease in *Nlgn3* protein with cerebral lysate (Fig. 1H), suggesting low Nlgn3 protein levels in cerebral astrocytes.

The partial decrease in Nlgn3 protein after astrocyte-specific deletion of *Nlgn3* raises the question of whether this decrease could be due to the Aldh1L1-Cre driver line used. To address this question, we used a tamoxifen-inducible GLAST-Cre/ER mouse line [47]. With this mouse line, we also observed that in the cerebellar cortex, tamoxifen induced Cre-mediated recombination in all Bergmann glia and a subset (~10%) of velate astrocytes, but not in neurons (Supplementary Fig. S1A–E). Consistent with these data, GLAST-Cre mediated deletion of *Nlgn3* induced a ~30% reduction of Nlgn3 protein levels in the cerebellar homogenate (Supplementary Fig. S1F). Thus, with two independent Cre lines, we demonstrated that Nlgn3 is enriched in cerebellar astrocytes.

### Astrocytic tagged Nlgn3 localizes to the cell body of astrocytes

**Fig. 2:**
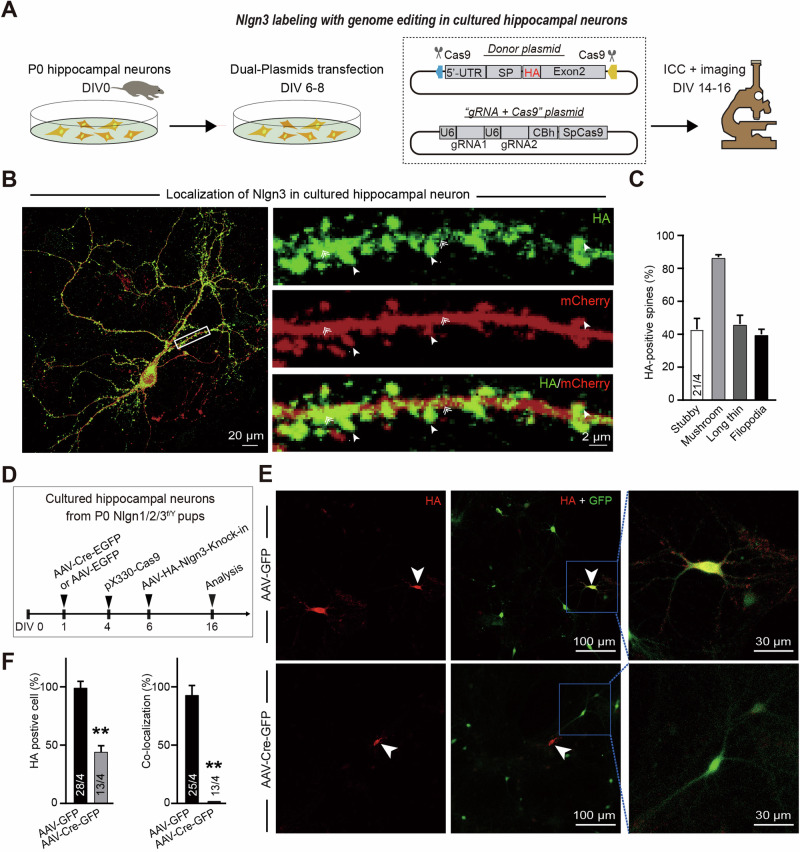
CRISPR/Cas9 mediated acute labeling of endogenous Nlgn3 with an HA tag in cultured hippocampal neurons as a proof of principle. **A** Experimental strategy for Nlgn3 labeling by genome editing in cultured hippocampus neurons. **B** Representative images showing the HA (green) signal of endogenous Nlgn3 in cultured hippocampus neurons, demonstrating that HA tagging does not disrupt the trafficking of Nlgn3 to synapses. **C** Distribution of HA (green) signal in different types of spines in cultured hippocampus neurons. **D** Procedures for Nlgn3 labeling by genome editing in cultured hippocampal neurons from newborn male Nlgn1/2/3 cKO mice with AAVs expressing GFP alone or together with Cre recombinase. **E** Representative images of hippocampal neurons infected with the AAV-Cre-GFP or AAV-GFP virus. **F** Quantification of the ratio of HA (red) positive cells and the co-staining ratio of the HA (red) with GFP with or without Cre from **E**. All data are shown as means ± SEM, and the numbers of cells/independent cultures analyzed are shown in the bar graphs by Welch’s *t*-test, *p < 0.05; **p < 0.01; ***p < 0.001.

Next, we studied the subcellular localization of cerebellar astrocytic Nlgn3. Since we were unable to find an *Nlgn3* antibody that worked in immunohistochemistry, we tagged genomic *Nlgn3* with an HA tag using a CRISPR/Cas9-mediated knock-in approach [48], thereby labeling endogenous Nlgn3. To develop and validate this approach, we first transfected primary cultured hippocampus neurons with plasmids containing two *Nlgn3* guide RNAs along with SpCas9 and HA-Nlgn3 donor DNA fragments (Fig. 2A). Partial tagging of endogenous Nlgn3 could be achieved using this approach, which allowed us to localize endogenous HA-tagged Nlgn3 in neurons. At DIV14-16 we imaged transfected neurons and found neurons with punctate signal enriched both in the dendrite shaft and in spines, especially in mushroom spines (Fig. 2B, C), consistent with previous functional studies on neuronal Nlgn3 both in excitatory and inhibitory synapses [2, 10, 49]. To confirm the specificity of our CRISPR-mediated HA tagging of endogenous *Nlgn3*, we cultured hippocampal neurons from Nlgn1/2/3 triple conditional knockout mice [10] and deleted the Nlgn genes by infecting the neurons with AAVs expressing Cre recombinase [50]. Subsequent CRISPR-mediated HA tagging of Nlgn3 in these neurons did not produce detectable punctate HA signals, demonstrating selective labeling of Nlgn3 in cultured hippocampal neurons (Fig. 2D–F).

**Fig. 3:**
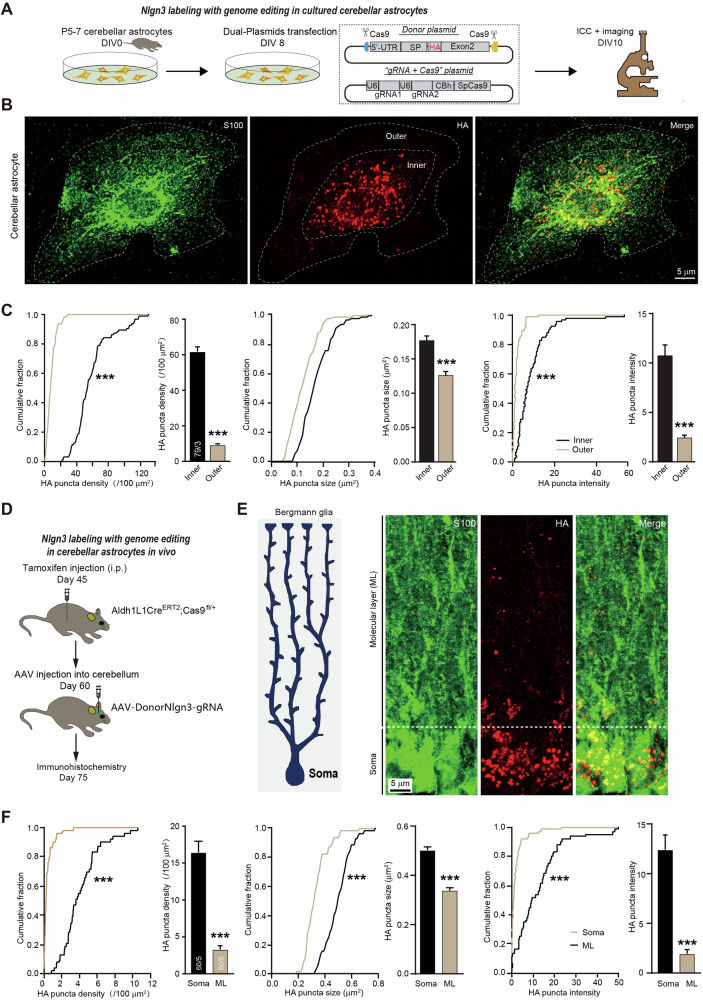
Endogenous astrocytic Nlgn3 visualized via acute CRISPR-mediated HA tagging is primarily located in the soma. **A** Experimental strategy for CRISPR-mediated acute HA-tagging of endogenous Nlgn3 in cultured cerebellar astrocytes. **B** Representative images showing immunostaining of HA (red) with S100 protein(green) in cerebellar astrocytes. **C** Cumulative and summary data for the HA puncta density, puncta size, and puncta intensity in the inner and outer region of the cerebellar astrocytes. **D** Flow chart illustrating in vivo tagging of Nlgn3 in Aldh1L1-Cre/ERT2; Cas9^fl/Y^ transgenic mice. **E** Representative images showing the immunostaining of HA with S100 in Soma and molecular layer. **F** Cumulative and summary data for the HA puncta density, puncta size, and puncta intensity in Soma and Molecular layer of the Bergmann glia in the cerebellum. All data are shown as means ± SEM, Statistical analysis was performed by Welch’s *t*-test (bar diagrams) or Kolmogorov–Smirnov test (cumulative distributions); *p < 0.05; **p < 0.01; ***p < 0.001. numbers of cells/Independent cultures (**C**) and cells/mice (**F**) analyzed are shown in the bar graphs.

Next, we labeled endogenous Nlgn3 with an HA tag in cultured cerebellar astrocytes. Immunofluorescence imaging revealed that Nlgn3 protein was highly enriched in the cell body of cultured cerebellar astrocytes where they form puncta, while little HA signal was detected in the distal processes of astrocytes (Fig. 3A–C, and Supplementary Fig. S2A). Analysis of subcellular localization of astrocytic Nlgn3 in cultured cerebellar astrocytes revealed that the majority of astrocytic Nlgn3 is in the cell organelles, including the endoplasmic reticulum, mitochondria, and Golgi (Supplementary Fig. S3).

To verify whether the unexpected somatic expression pattern of astrocytic Nlgn3 exists in vivo, we sought to HA-tag *Nlgn3* in cerebellar astrocytes in situ. We injected adenoviruses encoding guide RNAs and donor sequences into the cerebellar cortex of Aldh1L1-Cre/ERT2; Cas9^fl/+^ mice. The tamoxifen-induced expression of Cas9 in astrocytes allows us to selectively label Nlgn3 in astrocytes (Fig. 3D). Another AAV virus expressing EGFP was co-injected for the identification of injection sites. We waited for 2-3 weeks to allow time for genome editing and turnover/replacement of endogenous untagged Nlgn3 protein. In HA-positive cells, we found that the HA signal co-localized with astrocyte marker S100 protein signals (Fig. 3E), demonstrating selective labeling of Nlgn3 in cerebellar astrocytes. The detailed analysis demonstrated that the majority of Nlgn3 formed puncta on the cell body of cerebellar astrocytes (Fig. 3F, Supplementary Fig. S2B). To test the specificity of labeling of Nlgn3 in cerebellar astrocytes, we co-labeled the calbindin (labeling PC) with endogenous HA-Nlgn3 and revealed that the majority of Nlgn3 puncta formed within the BG area when compared to BG adjacent area (Supplementary Fig. S4A, B). We also found that *Nlgn3* is expressed in a subset of astrocytes in the deep cerebellar nuclei (Supplementary Fig. S4C, D). Together with data from cultured astrocytes, we thus demonstrate that HA-tagged endogenous Nlgn3 is enriched in cerebellar astrocytes and predominantly expressed in the cell body of Bergman glia.

### Astrocytic *Nlgn3* is dispensable for synaptic transmission

**Fig. 4:**
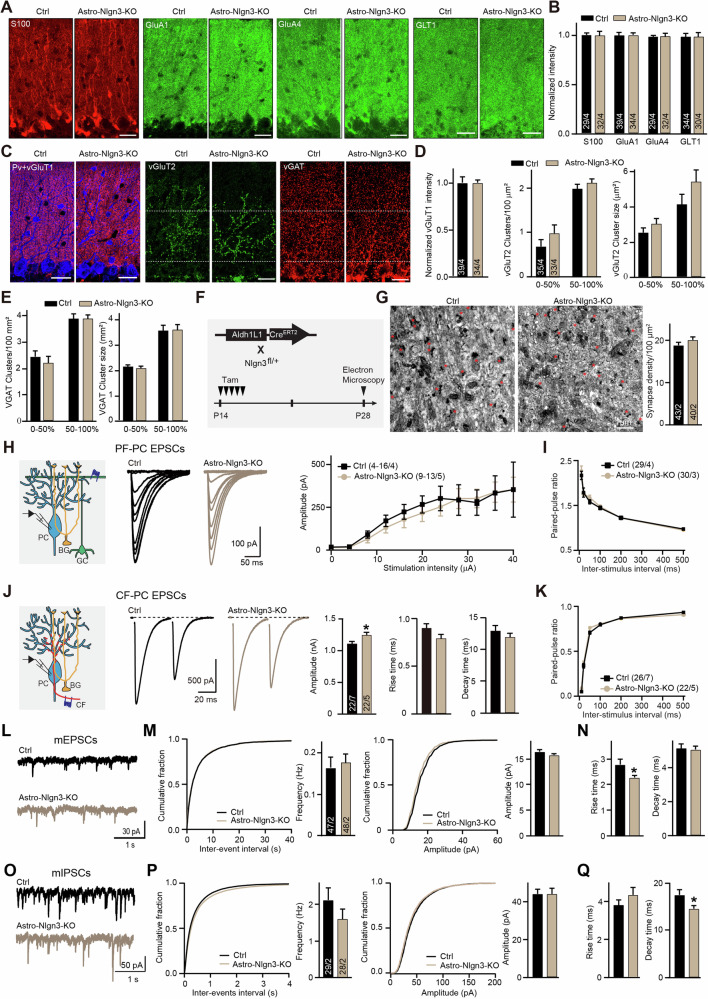
Astrocytic Nlgn3 is dispensable for maintaining synapse numbers and for synaptic transmission in the cerebellar cortex. **A** Representative IHC images showing the distribution of S100 protein, GluA1, GluA4, and GLT1 in Aldh1L1-Nlgn3-KO mice and littermate control in the cerebellar cortex. Animals of control and Aldh1L1-Nlgn3-KO mice were injected with tamoxifen between P14 and P18 and further analysis was performed between P24 and P28 in the figure. **B** Summary graphs of the signals for various markers in cerebellar sections labeled with antibodies as described in **A**. **C** Representative images showing vGluT2, vGAT, and vGluT1 labeling in Aldh1L1-Nlgn3-KO mice and littermate control cerebellar cortex. Scale bars in **A** and **C**, 20 μm. **D**, **E** Summary graphs of cerebellar sections labeled with antibodies in **C**. **F**, **G** Representative EM images and summary graph for synapse density in the upper third of molecular layer from Aldh1L1-Nlgn3-KO mice and littermate control cerebellar cortex. PF-PC synapses are indicated with red asterisks. Scale bar, 1 μm. **H**, **I** Representative traces and summary graphs for the input-output curve (**H**) and paired-pulse ratio (**I**) of parallel fibers evoked EPSCs in Purkinje cells. **J**, **K** Representative traces and summary graphs of climbing fiber-EPSCs recorded in Purkinje cells. The climbing fibers were activated by paired stimulation with an interval of 50 ms. The different inter-stimulus intervals for paired-pulses ratio recording were indicated. **L**-**N** Sample traces (**L**), cumulative plots (**M**), and kinetics (**N**) of mEPSC recorded from P24-P28 Purkinje cells. **O**–**Q** Sample traces, cumulative plots, and kinetics of mIPSC recorded from P24-P28 Purkinje cells. All data are shown as means ± SEM, statistical analysis was performed by Welch’s *t*-test (bar diagrams) or Kolmogorov–Smirnov test (cumulative distributions); numbers of sections or neurons/mice analyzed are shown in the bar graphs, *p < 0.05.

The robust expression of *Nlgn3* in cerebellar astrocytes prompted us to examine its role in sculpting the shape of astrocytes and the number and function of parallel-fiber synapses. To morphologically examine such a role, we double-labeled cryo-sections from littermate control and Astro-Nlgn3-KO mice with antibodies to S100 protein, GluAs (Bergmann glia-enriched isoforms), vGluT1, vGluT2, and vGAT (markers for excitatory and inhibitory presynaptic terminals, respectively). The deletion of astrocytic *Nlgn3* did not alter any parameters we measured in the cerebellar cortex (Fig. 4A–E), suggesting that the deletion of astrocytic *Nlgn3* did not affect the gross morphology of cerebellar astrocytes or synapse numbers. Expectedly, the deletion of astrocytic Nlgn3 did not affect the PF-PC synapse number at the ultrastructural level with electron-microscopy (Fig. 4F, G).

To test for a possible role of astrocytic *Nlgn3* in regulating synaptic transmission, we monitored excitatory and inhibitory synaptic transmission in Purkinje cells by whole-cell patch-clamp recordings in acute cerebellar slices from control and Astro-Nlgn3-KO mice at P24-P28, which is 10-14 days after Cre-activation. The astrocytic *Nlgn3* deletion produced a modest increase in the amplitude of EPSCs evoked by climbing-fiber stimulation without affecting parallel-fiber EPSCs (Fig. 4H–K), miniature EPSCs (mEPSCs) (Fig. 4L–N) and miniature IPSCs (mIPSCs) (Fig. 4O–Q). Viewed together, these data suggest that the astrocytic *Nlgn3* deletion does not significantly affect the number and properties of synapses in the cerebellar cortex. The phenotypes obtained from Astro-Nlgn3-KO mice are notably different from those observed for deletions of AMPARs or glutamate transporters from Bergmann glia [43, 44], implying that astrocytic *Nlgn3* is unlikely to directly or indirectly cluster/stabilize AMPARs and glutamate transporters that are part of tripartite synapses in cerebellar astrocytes.

By using immunohistochemistry and electrophysiological recordings, we examine the effect of astrocytic *Nlgn3* deletion on synapse number and properties in the layer II/III of somatosensory cortex from control and Astro-Nlgn3-KO mice at P24-P28 (10-14 days after Cre-activation). We found that deletion of astrocytic *Nlgn3* does not affect synapse number, synaptic transmission, and astrocyte morphology (Supplementary Fig. S5). Consistent with our data, a recent study demonstrated that selective deletion of Nlgn1/2/3 in astrocytes does not alter synapse number, synaptic transmission, and astrocyte morphology in the cortex [30]. Not surprisingly, the cell density both in the cerebellum and the somatosensory cortex is intact in Astro-Nlgn3-KO mice (Supplementary Fig. S6).

### Spatial mapping of gene expression of cerebellar astrocytes

**Fig. 5:**
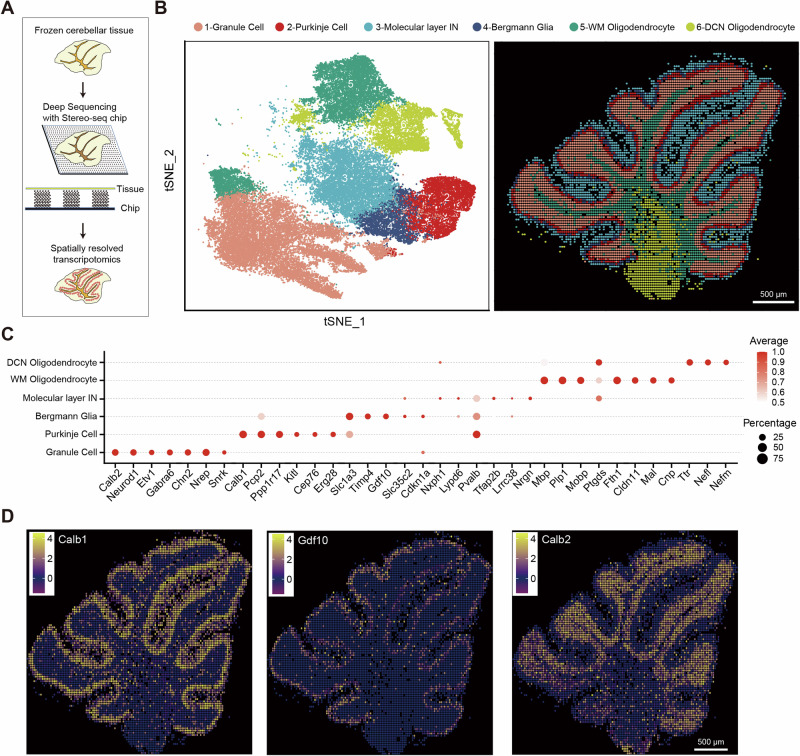
Spatial transcriptomic-based reconstruction of cerebellar gene expression. **A** Schematic of Stereo-seq experiments applied to the cerebellum. Mouse cerebellar samples were collected and prepared by 10 µm thickness frozen sections. Then, in situ RNA capture of the cerebellum was performed on the 1 cm^2^ Stereo-seq chips. Followed by RNA reverse transcription, cDNA amplification, library construction, and sequencing. **B** UMAP plot (left) for 46,654 spots colored by 6 cell types, and spatial visualization (right) of 6 cell types in the cerebellum. **C** Bubble plot showing the expression level of specific markers defining major cell types in the cerebellum. Dot size represents the fraction of expressing cells. The color indicates the Z score scaled gene expression levels. **D** In situ display of *Calb1* (a marker of Purkinje cell), *Gdf10* (a marker of Bergmann glia), and *Calb2* (a marker of granule cell). Heat maps indicate the expression levels of indicated genes. The color indicates the Z score scaled gene expression levels.

The observation of the somatic localization of astrocytic Nlgn3 protein prompted us to test its potential role in gene expression. To achieve this, we adopted the Stereo-seq method [51, 52], a recently developed spatial transcriptomic approach, that enables us to analyze the impact of *Nlgn3* on gene expression in cryosections of the cerebellum. Brain sections of the cerebellar cortex from Astro-Nlgn3-KO and littermate control mice were embedded to prepare sagittal cryosections with a 10 μm thickness, which roughly corresponds to a single cell layer. We then applied high-resolution Stereo-seq chips to capture transcriptome in situ (Fig. 5A). To minimize incomplete sampling of cell types and batch effects, we collected brain sections from 3 Astro-Nlgn3-KO and 3 male littermate control mice for Stereo-seq library preparation. In total, we collected transcriptomic data for 46,654 bin 50 spots from six Stereo-seq slices, with a size of 25 × 25 μm per spot. As expected, we observed more refined clustering with increased cluster numbers and higher accuracy (Fig. 5B). The same clusters were identified in each sample, and the number of spots in the clusters was similar (Supplementary Fig. S7A, Supplementary Table S1). Thus, our use of Stereo-seq enabled us to construct a high-quality spatial transcriptome of the cerebellum.

We performed unsupervised t-distributed stochastic neighbor embedding (tSNE) analyses to cluster all the bin 50 spots, revealing spatial heterogeneity of gene expression in the cerebellar slices (Fig. 5B). The obtained transcriptomic configurations matched the localization of major anatomic structures. We assigned each cluster to a known cell type identity based on the expression of specific molecular markers that are known to characterize morphological, histological, and/or functional features, including granule cell markers (*Calb2*^*+*^*, Neurod1*^*+*^*, Etv1*^*+*^*, Gabra6*^*+*^*, Chn2*^*+*^*, Nrep*^*+*^*, Snrk*^*+*^), Purkinje cell markers (*Calb1*^*+*^*, Pcp2*^*+*^*, Ppp1r17*^*+*^*, Kitl*^*+*^*, Cep76*^*+*^*, Erg28*^*+*^), Bergmann glia markers (*Slc1a3*^*+*^*, Timp4*^*+*^*, Gdf10*^*+*^*, Slc35c2*^*+*^*, Cdkn1a*^*+*^), molecular layer interneurons markers (*Nxph1*^*+*^*, Lypd6*^*+*^*, Pvalb*^*+*^*, Tfap2b*^*+*^*, Lrrc38*^*+*^*, Nrgn*^*+*^), white matter oligodendrocyte markers (*Mbp*^*+*^*, Plp1*^*+*^*, Mobp*^*+*^*, Ptgds*^*+*^*, Fth1*^*+*^*, Cldn11*^*+*^*, Mal*^*+*^*, Cnp*^*+*^), and deep cerebellar nuclei oligodendrocyte markers (*Ttr*^*+*^*, Nefl*^*+*^*, Nefm*^*+*^*, Ptgds*^*+*^) (Fig. 5C). These annotations were consistent with layer-specific localizations of marker genes in the Allen Brain Atlas. Moreover, we identified cell-type-specific genes, such as *Chn2*, *Nrep*, and *Snrk* of granule cells and *Kitl*, *Cep76*, and *Erg28* of Purkinje cells, that had not been previously reported as marker genes. In situ expression of these genes represented the anatomy of the cerebellum (Fig. 5B–D). Notably, we found that the same cell type could contain multiple clusters defined by differentially expressed genes (DEGs), suggesting heterogeneity within the same cell type [53] (Fig. 5C).

### Astrocyte-specific Nlgn3 knockout results in non-cell autonomous effects on transcription across the brain

**Fig. 6:**
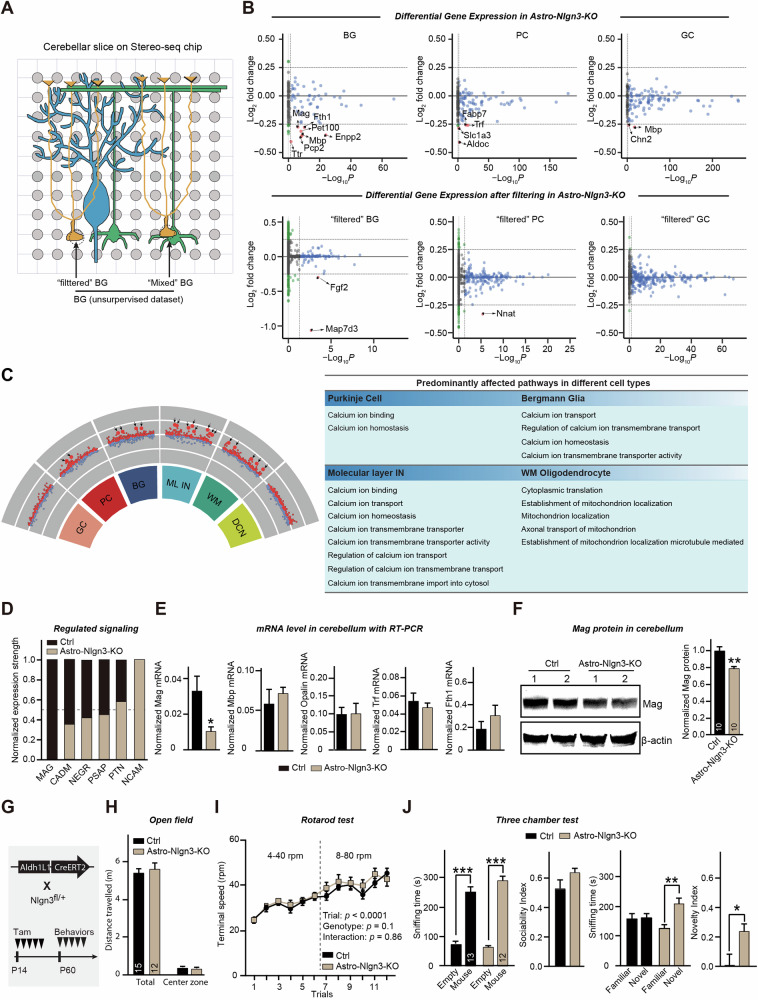
Astrocytic Nlgn3 regulates transcription in the cerebellum. **A** Schematic of Stereo-seq experiments applied to the cerebellum. **B** Volcano plots showed differential gene expression (DGE) in Bergmann glia, Purkinje cells, and Granule cells with the unsupervised dataset. To improve the precision of mRNA detection in different cell types, we further “filtered” unsupervised datasets using marker genes, resulting in the identification of “filtered” Bergmann glia, “filtered” Purkinje cells, and “filtered” Granule cells. **C** Quantitative analysis of pathway activity. The inner circle represents the cell types, and the outer circle represents the differential pathways. In the gray block of the outer circle, the three white lines from the inside to the outside are −0.5, 0, and 0.5. Up-regulated pathways are indicated by red dots, whereas down-regulated pathways are denoted by blue dots. All pathways with |log2 fold change|>0.25 and adjusted *p*-value < 0.05 are marked with black arrows and the predominantly affected pathways are summarized in the table on the right. **D** Normalized expression strength of cell communication signaling pathways among Bergmann glia, Purkinje cell, Granule cell, and molecular layer interneurons. **E** RT-qPCR analysis of selected genes in the cerebellum from P24 and P28 control and Aldh1L1-Nlgn3-KO mice. **F** Representative quantitative immunoblots and summary graphs of Mag protein in the cerebellar tissue lysate of P24 Aldh1L1-Nlgn3-KO mice and littermate control. **G** Animals of control and Aldh1L1-Nlgn3-KO were injected with tamoxifen between P14 and P18 and further behavior analysis was between 2-3 months. **H–J** Quantitative analysis with open field test (Distance (total vs Center zone): F_1,50_ = 576.5, *p* < 0.0001; genotype: F_1,50_ = 0.155, *p* = 0.6955; distance Χ genotype interaction: F_1,50_ = 0.3409, *p* = 0.562; two-way ANOVA) (**H**), accelerating rotarod test (trial: F_11, 300_ = 18.43, *p* < 0.0001; genotype: F_1, 300_ = 2.2724, *p* = 0.1); trial Χ genotype interaction: F_11, 300_ = 0.5549, *p* = 0.8644; two-way ANOVA) (**I**), and three-chamber test (Social ability: sniffing (empty vs. mouse): F_1,46_ = 271.9, *p* < 0.0001; genotype: F_1,46_ = 1.236, *p* = 0.272; sniffing Χ genotype interaction: F_1,46_ = 4.133, *p* = 0.0479. Both genotypes sniff more with a mouse than the empty cup: Ctrl (*p* < 0.0001) and Astro-Nlgn3-KO (*p* < 0.0001); Social novelty: sniffing (familiar vs. novel): F_1,46_ = 7.384, *p* = 0.0092; genotype: F_1,46_ = 0.2677, *p* = 0.6074, sniffing Χ genotype interaction: F_1,46_ = 6.534, *p* = 0.014). Astro-Nlgn3-KO mice sniff more with the novel mouse than the familiar one: Ctrl (*p* = 0.9078) and Astro-Nlgn3-KO (*p* = 0.0013); two-way ANOVA with the Holm-Sidak post hoc test; Data analyzed in sociability index and novelty index are shown by Welch’s *t*-test) (**J**). All data are shown as means ± SEM, numbers of mice analyzed are shown in the bar graphs by Welch’s *t* test (**E**, **F**) or two-way ANOVA with or without the Holm-Sidak post hoc test (**H**–**J**), *p < 0.05; **p < 0.01; ***p < 0.001.

After performing spatial transcriptomics on cerebellar sections, we analyzed the impact of astrocytic *Nlgn3* deletions on gene expression (Fig. 6A). We found a modest yet statistically significant decline in gene expression in most cerebellar cell types, including 7 genes in Bergmann glia, 4 genes in Purkinje cells, and 2 genes in granule cells, after the astrocytic *Nlgn3* deletion. DEGs in Bergmann glia enriched in myelination, including *Mag, Fth1, Mbp*, and *Ttr* (Fig. 6B, Supplementay Table S2). The size of 25 × 25 μm per spot might touch more than one cell type, we refined our analysis by filtering the datasets based on prior knowledge (different cell types have different cell markers) to isolate clusters of specific cell types (Fig. 6A, B). We obtained clusters of “filtered Bergmann glia” (*Mbp/Mag/Enpp2/Plp1/Opalin/Vamp5/Pcp2* negative Bergmann Glia), “filtered Purkinje cells” (*Mbp/Mag/Enpp2/Plp1/Opalin/Vamp5/Slc1a3* negative Purkinje Cell), and “filtered Granule cells” (*Mbp/Mag/Enpp2/Plp1/Opalin/Vamp5* negative Granule cell), (Fig. 6A, B, Supplementay Fig. S7A–C), reveal the DEGs in pure Bergmann glia, pure Purkinje cells, and pure granule cells. We found that only a few genes are altered in the filtered datasets (Fig. 6B, Supplementay Table S3), indicating that astrocytic Nlgn3 has a cell non-autonomous role in gene regulation, affecting the expression of mRNA in other cerebellar cell types.

Nlgn3 might regulate calcium balance and participate in calcium-regulated cellular processes, as suggested in an in vitro study [54]. Therefore, those organelles-localized Nlgn3 might regulate calcium homeostasis and then regulate other biological processes, including gene regulation in the astrocytes. To test this hypothesis, we quantitatively analyzed the activity changes of calcium ion and organelle localization-related pathways after *Nlgn3* knockout. Based on the thresholds of |log2 fold change|>0.25 and adjusted *p*-value < 0.05, we identified 4 up-regulated pathways in Bergmann glia, 2 up-regulated pathways in Purkinje cells, 8 up-regulated pathways in molecular layer IN, and 4 up-regulated pathways in WM Oligodendrocytes. In Bergmann glia, Purkinje cells, and molecular layer IN, calcium-associated signaling pathways were significantly activated (including calcium ion transmembrane transport), implying a change in cell communication between neurons and glial cells (Fig. 6C).

We further investigated the role of astrocytic Nlgn3 in cell-cell communication in the cerebellum using CellChat [55]. We found that the Myelin‐associated glycoprotein (MAG) pathway was selectively active in the wildtype cerebellum, while the neural cell adhesion molecule (NCAM) pathway was active in the Astro-Nlgn3-KO cerebellum, indicating a possible shift in the active pathways (Fig. 6D). We also performed RT-PCR to measure expression levels of *Mag, Mbp, Opalin, Trf*, and *Fth1* in the entire cerebellum. The mRNA expression and protein levels of *Mag* are significantly down-regulated in Nlgn3 deletion mice, implying functional impairment in the formation and maintenance of myelin sheaths in the cerebellum (Fig. 6E, F). However, Nlgn3 does not directly bind to MAG when overexpressed in HEK293 cells with cell aggregation assay (Supplementary Fig. S8), excluding the possibility that transcellular interaction between MAG and Nlgn3 mediates the regulation of gene expression.

To test whether the astrocytic *Nlgn3* deletion on gene expression beyond the cerebellum, we extended our analysis to include existing transcriptomic data from the V1 cortical area of the same batches of brain samples (Supplementary Fig. S9, Supplementary Table S4). We identified 15 DEGs after the deletion of astrocytic Nlgn3 deletion and, interestingly, those DEGs in the V1 cortex are distinct from the DEGs observed in the cerebellum and are enriched in functions associated with autism spectrum disorder (Supplementary Fig. S9E). Since the Nlgn3 level in cortical astrocytes is low, it is likely that the DEGs in the cortex after astrocytic Nlgn3 deletion may be the consequence of behavioral changes or other system-level phenomena. A subtle contribute of genetic background cannot totally be excluded.

To investigate the effect of astrocytic *Nlgn3* deletion on mouse behavior, we performed open-field and accelerating rotarod tests to assess motor activity and coordination. We found no significant differences in these behaviors between the control and Astro-Nlgn3-KO mice. However, in the three-chamber test, which assesses social behavior, we identified an enhanced social novelty phenotype without changes in sociability (Fig. 6G–J). Our control mice do not show a social novelty preference, potentially due to strain background issues. Whether those brain regions that are known essential for social novelty (e.g. Hypothalamus and the olfactory bulb), contribute to the social novelty phenotype warrants further studies. Overall, our results suggest that astrocytic Nlgn3 may contribute to the regulation of gene expression and may thereby affect mouse behavior.

## Discussion

Here, we have studied the localization and function of astrocytic Nlgn3 in the cerebellum. We have shown that Nlgn3 is abundant in cerebellar astrocytes that contain ~40% of the total Nlgn3 protein of the cerebellum. In cerebellar astrocytes, Nlgn3 is localized to the cell body of these cells and not to the fine distal perisynaptic processes (Figs. 1–3). We also revealed that the deletion of astrocytic *Nlgn3* modestly results in the alteration of gene expression and mouse behavior without affecting the number and function of synapses in the cerebellum (Figs. 4–6). If generally applicable, this conclusion suggests that astrocytic neuroligins are intrinsically different from neuronal neuroligins and that astrocytic neuroligins are not the building block of the tripartite synapse but likely modulate gene expression.

The functional sites of neuronal neuroligins can be at the postsynaptic sites, extrasynaptic sites [13], or partially at the cell body [56]. Using a genetic mouse line with HA-tagged Nlgn1, a sizable fraction of Nlgn1 in the cerebellum localizes in the Bergmann glia [57]. One study suggested that Nlgn2 in cerebral astrocytes is essential for astrocyte morphology, is localized to excitatory synapses, and is essential for excitatory synapse formation [58], but many other studies have detected Nlgn2 protein only in inhibitory synapses [59, 60] and deletions of *Nlgn2* from all cells in the brain does not cause a decrease in excitatory synapse numbers [18, 20, 61]. This discrepancy has created significant controversy which remains unresolved. Interestingly, in our experiments we did not observe a significant reduction of Nlgn3 protein levels outside of the cerebellum in Astro-Nlgn3-KO mice, suggesting that Nlgn3 protein is not a major component of astrocytes outside of the cerebellum.

Our arguably most important result is that the deletion of *Nlgn3* from cerebellar astrocytes in the developing cerebellum has virtually no effect on synapse numbers or synaptic transmission in the cerebellar cortex (Fig. 4). The phenotype of the *Nlgn3* deletion in cerebellar astrocytes differs substantially from that obtained by genetic manipulations of astrocytic proteins enriched in tripartite synapses, such as AMPARs and glutamate transporters. Overexpression of Ca^2+^-impermeable AMPARs [42] or deletions of AMPARs [43] or glutamate transporters [44] caused a retraction of the Bergman glia processes that cover parallel-fiber synapses, increased synaptic strength, and similarly affected both parallel-fiber and climbing-fiber synapses. Astrocytic AMPARs or glutamate transporters are enriched in the astrocyte processes, while astrocytic Nlgn3 is primarily located in the cell body of cerebellar astrocytes (Fig. 3). Together, those results further strengthen the idea that the processes and the cell body of cerebellar astrocytes form different functional compartments via different molecular machinery.

With spatial stereo-seq, we mapped the gene expression patterns of the cerebellum as a function of the astrocytic *Nlgn3* deletion. We found broad gene expression changes in multiple cell types both in the cerebellum and in the cortex after the deletion of astrocytic *Nlgn3*. We found the downregulation of Myelin-related proteins, including MAG. However, MAG does not directly bind to Nlgn3 with cell aggregation assay, suggesting surface Nlgn3 is less likely to modulate gene expression via membrane protein MAG. Currently, the detailed mechanisms of cerebellar astrocytic Nlgn3 on gene expression are still unknown. Since astrocytic Nlgn3 is predominantly located in the organelles of cerebellar astrocytes, astrocytic Nlgn3 likely regulates gene expression via recruiting non-canonical binding partners [54, 62] that mediate signal transduction, e.g. calcium signaling [54], consistent with our analysis of astrocytic Nlgn3 on calcium homeostasis (Fig. 6C). These alterations in gene expression might influence the function of astrocytes [63–70], thereby impacting mouse behavior [71]. The minimal social novelty preference (likely due to strain background difference) observed in the control mice might be one limitation of the current behavior study. Interestingly, we also observed DEGs in the cortex. Considering the low expression astrocytic Nlgn3 level and distinct gene clusters of DEG, the DEGs observed in the cortex are likely due to a result of behavioral changes Astro-Nlgn3-KO animals display, although this warrants further investigation.

The role of astrocytic *Nlgn3* is different from that of astrocytic *Nlgn2* by the earlier report which suggested an essential role of astrocytic *Nlgn2* on astrocyte morphology and synapse function [58]. Importantly, complete deletion of astrocytic neuroligins (Nlgn1/2/3, Note: *Nlgn4* is minimally expressed in non-neuronal cells) does not affect synapse number, synapse function, and astrocyte morphology in the cortex [30]. The question arises of how our findings may relate to *NLGN3* mutations observed in patients with autism spectrum disorders (ASDs) [72, 73]. Considering this possibility in exploring the pathogenesis of ASDs will be important on a global scale if we ever want to understand how pervasive gene mutations predispose the brain to neuropsychiatric disorders.

## Supplementary information

Supplemental data

## Data Availability

All data are available in the main text or supplementary materials. We uploaded the raw data of spatial transcriptomic to Spatial TranscriptOmics DataBase (STOmicsDB) (https://db.cngb.org/stomics/) with accession number STT0000040. Further information and requests for resources and reagents should be directed to and will be fulfilled by the Lead Contact, Bo Zhang (zbo@pku.edu.cn or zbo@szbl.ac.cn).
