# Supplementary material for: Astrocytic Neuroligin-3 influences gene expression and social behavior, but is dispensable for synapse number
Source: Mol Psychiatry. 2024 Jul 13;30(1):84–96. doi: 10.1038/s41380-024-02659-6 (PMC11649564; doi:10.1038/s41380-024-02659-6)
Supplement: Supplementary file 1 — Supplemental data [file 41380_2024_2659_MOESM1_ESM.docx]

Supplementary Materials for

**Astrocytic Neuroligin-3 influences gene expression and social behavior, but is dispensable for synapse number**

Liming Qin^1,2,†^, Zhili Liu^3,4,†^, Sile Guo^1,2^, Ying Han^1,2^, Xiankun Wang^1,2^, Wen Ren^1,2^, Jiewen Chen^1,2^, Hefu Zhen^3^, Chao Nie^3^, Ke-Ke Xing^5^, Tao Chen^5^, Thomas C. Südhof ^6,*^, Yuzhe Sun^3,7,8,*^, Bo Zhang^1,2,*, §^

Short title: Neuroligin-3 Function in cerebellar astrocytes

^1^ School of Chemical Biology and Biotechnology, Peking University Shenzhen Graduate School, Shenzhen 518055, China;

^2^ Institute of Neurological and Psychiatric Disorders, Shenzhen Bay Laboratory, Shenzhen 518132, China;

^3^ BGI Research, Shenzhen 518083, China;

^4^ Department of Obstetrics and Gynaecology, The Chinese University of Hong Kong, Hong Kong, China;

^5^ Department of Anatomy and K.K. Leung Brain Research Centre, Fourth Military Medical University, Xi’an 710032, China;

## ^6^ Department of molecular and cellular physiology, Howard Hughes Medical Institute, Stanford University School of Medicine, United States, 94043;

^7^ BGI Research, Beijing 102601, China;

^8^ Shenzhen Key Laboratory of Neurogenomics, BGI-Shenzhen, Shenzhen, 518120, China;

## ^†^Equal contribution

## ^§^ Lead contact

^*^Correspondence: [zbo@pku.edu.cn](mailto:zbo@pku.edu.cn) (B. Z.), [yzsun@connect.hku.hk](mailto:yzsun@connect.hku.hk) (Y. S.). or [tcs1@stanford.edu](mailto:tcs1@stanford.edu) (T.C.S.)

**Supplemental Methods, Tables, and Figures**

**Tamoxifen injection**: Tamoxifen (T5648, Sigma-Aldrich) was prepared weekly by dissolving in 90% sunflower seed oil/10% ethanol solution by bath sonication for 20~30 minutes with intermittent vortexing. The final concentration of tamoxifen was 10 mg/ml. Mice at P14-P18 were injected intraperitoneally (i.p.) with 80 mg/kg of tamoxifen once daily for 5 days and analyzed 10 days or 6 weeks later. Mice exhibiting discomfort during tamoxifen treatments (e.g. decreased mobility, increased tremors, dehydration, rough coat, or gasping) were excluded for further analysis.

**DNA constructs/plasmids:** The guides were created using the Benchling CRISPR tool (http://www.benchling.com). pX330 and pMiniT vectors were kindly gifted by Dr. Huaqiang Fang. For the dual-promotor guide RNA expression vectors, a pair of complementary 20 bp oligos for each guide RNA sequence was annealed and cloned into pX330 separately. Sequencing confirmed the constructs. To generate one vector with the two guides, both dual-U6 promoter and single guide RNA expression cassette were amplified separately from gRNA1- Nlgn3-pX330 and gRNA2-Nlgn3-pX330 plasmid by primers. Then these fragments can be assembled by the In-Fusion® HD Cloning Kit (PT5162-1, Clontech). All the CRISPR/Cas9 guide RNA sequences and PAM sequences used in this study are provided below:

gRNA1-NLGN3-F: 5’-caccGAAGGGAGCCCTCTTTCGAAG-3’;

gRNA1-NLGN3 -R: 5’- aaacCTTCGAAAGAGGGCTCCCTTC-3’;

gRNA2- NLGN3-F: 5’-caccGTGACACTGATGCCAGAAGG-3’;

gRNA2-NLGN3 -R: 5’- aaacCCTTCTGGCATCAGTGTCAC-3’.

For the construction of each knock-in donor plasmid, two DNA fragments were generated by PCR amplification from gDNA plasmids using PrimeSTAR® Max DNA Polymerase (Cat. #R045A, TaKaRa). Fragment 1 included the genomic DNA between guide 1 and the KI site, with a flipped guide 2 (with PAM) at its 5’-end; fragment 2 included the genomic DNA between the KI site and guide 2, with a flipped guide 1 (with PAM) at its 3’-end. These two DNA fragments were integrated into a vector backbone (pMiniT vector) by the In-Fusion® HD Cloning Kit (PT5162-1, Clontech). All knock-in donors’ sequences were confirmed by pMiniT forward or reverse sequencing primers. The PCR primers utilized in the construction of knock-in donors were as follows:

pMiniT-Nlgn3-F: 5’- CTTCGAAAGAGGGCTCCCTTCCATCATGGTTTTTACCT CC -3’; pMiniT-Nlgn3 -R: 5’- ACACTGATGCCAGAAGGGGGCCCTGATAATAA TGACGTCAG -3’ ;

Nlgn3-pMiniT-F: 5’-AGG TAA AAA CCA TGA TGG AAG GGA GCC CTC TTT CGA AGT GGT CAC ACA TGG GAG CTC GG -3’;

Nlgn3-pMiniT-R: 5’- ACG TCA TTA TTA TCA GGG CCC CCT TCT GGC ATC AGT GTC ACT GGT GCT CGG AAG ACC TG -3’ ;

HA-Nlgn3-pMiniT-F: 5’- GTA ATC TGG AAC ATC GTA TGG GTA TCC ACC GGT ACTG GCC CTC AGC ACC -3’;

HA-Nlgn3-pMiniT-R: 5’- CAT ACG ATG TTC CAG ATT ACG CTG GAG GTC AGG CCC CGG CAC CCA CAG T -3’

**AAV design and production:** The dual-U6 promoter expression cassette and the donor were amplified separately from the gRNA1-gRNA2-Nlgn3-pX330 plasmid and corresponding HA-Nlgn3-pMiniT donor plasmid. These two fragments were integrated into pAAV backbones with the In-Fusion® HD Cloning Kit (PT5162-1, Clontech). The resulting clones were confirmed by sequencing. The pAAV backbones are gifted by Dr. Huangqiang Fang. AAVs were packaged with serotypes 2/9 and were generated by Shanghai Taitool Bioscience Co., LTD, China.

**Virus injection surgery:** Mice were anesthetized with 1% pentobarbital (Sigma, USA), the skin above the skull was cleaned with ethanol wipes and prepared by removing the hair, and then fixed in a stereotaxic frame (RWD, China). A surgical incision was made to expose the skull, followed by a craniotomy to expose the brain surface. The virus was delivered using a glass capillary attached to a stereotaxic injector at a rate of 100 nL/min; After each injection, the pipette was kept in place for 5 minutes before either being raised to the next injection depth or slowly removed from the brain. The incision was sutured (sh-jinhuan). Animals were placed in a heated cage with access to softened food for recovery, 3 weeks of recovery was allowed for optimal viral expression. To target the cerebellum, the following stereotaxic coordinates and injection volumes were used, relative to bregma and pia. Cerebellum AP: -6.1, ML: + 1.0, Z: -2.0 (500 nl per site). Both viruses were not diluted before injection and had the following titers: AAV 2/9-HA-Nlgn3-knock-in (titer 1.42x10^13^Vg/ml, Shanghai Taitool Bioscience Co., LTD, Shanghai, China) and AAV 5-CAG-EGFP (titer: 1.68x10^13^Vg/ml, Shanghai Genechem Co., LTD, Shanghai, China).

For the dual virus experiments, AAV2/9-HA-Nlgn3-knock-in (titer 1.42x10^13^Vg/ml) was mixed 1:1 with AAV5-CAG-EGFP (titer 1.68x10^13^Vg/ml) and injected into the hemisphere of P60 Aldh1L1-Cre/ERT2; Cas 9^fl/Y^ transgenic mice (500 nl per site), Cas9 expression in astrocytes were induced after tamoxifen injection at P45 for 5 days.

**Primary cell cultures and transfection:** Primary cultures of cerebellar astrocytes were isolated from P5-P7 of C57BL/6J pups (both genders). Cortical and cerebellar tissue were dissected, treated with 0.125% trypsin, and dissociated into single cells by gentle trituration. Cells were resuspended in DMEM containing 10% FBS (Gbico), and then plate the cells in a 25-cm^2^ flask precoated with poly-D-lysine (PDL). Cultures were maintained in a humidified incubator at 37℃ with 5% CO2 for 7-10 days and media were changed every 3 days. Astrocytes can be purified from the mixed glia culture. Mixed glia cultures were shaken at 200 rpm overnight at 37℃ to dislodge other glia cells attached to the astrocyte layer. After medium replacement, astrocytes were obtained by trypsinization (0.125% trypsin, 5min, 37℃) and transferred to the glass coverslips precoated with PDL at a low density. When the cells' density reached 70%, they were transfected with dual plasmid DNA (gRNA1-gRNA2-Nlgn3-pX330 and HA-Nlgn3-pMiniT) using the calcium phosphate precipitation method (Cat#631312, Clontech) and analyzed 24-48h after transfection.

Primary cultures of hippocampal neurons were isolated from P0 of C57BL/6J pups (both genders). The hippocampus was dissected, treated with 0.125% trypsin, and dissociated into single cells by gentle trituration. Cells were resuspended in DMEM containing 10% F12 (Invitrogen) and 10% FBS (Gbico), and then seeded at 75.000 cells/ml on glass coverslips (8 x 8 cm) precoated with poly-D-lysine (PDL). Cultures were maintained in a humidified incubator at 37℃ with 5% CO2. On the second day, feed the cultures by replacing half of the volume with a neuronal maintenance medium (Neurobasal/B27/Glutamax). Replace half of the neuronal maintenance medium every 3 days. Cells were transfected with dual plasmid DNA (gRNA1-gRNA2-Nlgn3-pX330 and HA-Nlgn3-pMiniT) at DIV 6-8, using the calcium phosphate precipitation method (Cat#631312, Clontech) and analyzed at DIV 14-16 after transfection.

**Definition for inner and outer of astrocytes**

Cultured cerebellar astrocytes were labeled with S100, which is found diffusely in cytoplasm, associated membranes, and certain cytoskeleton elements. We traced their extended fine process to reveal the cell boundary (area of the whole cell, A_whole_); In the intracellularly perinucleus area, which was highly filled with radially S100 signal was arbitrarily defined as an inner area, and the outer area = area of whole cell- area of inner (A_outer_=A_whole_-A_Inner_).

**Classification of spines**

Dendritic spines were classified as described previously[1, 2]. Traditionally dendritic spines are grouped into four fixed classes according to their morphological features: mushroom, thin, stubby, and filopodia. Mushroom spines have a large head and a small neck, the diameter of the spine head is ≥1.5× the spine neck diameter (dh/dn≥1.5), separating them from a dendrite; Thin spines have a similar structure to the mushroom spines, but their spine head and neck are nearly equal, and the spine length is greater than spine width (dh/dn< 1.5, L/dh ≥2); Stubby spines typically do not have a neck, the ratio of the head and neck is<1.5, and spine head diameter is significantly longer than the spine length (dh/dn<1.5, L/dh<2); Filopodia are long, thin dendritic membrane protrusions without a clear head. The images were acquired with a Zeiss LSM980 with 63 x oil objective (PlanApo, NA1.4). Only images with sufficient quality and identification of spine shapes were included in the classification. Spine measurements were performed using Imaris software.

**Cell aggregation:** Cell aggregation experiments were carried out as described [3]. Cultured HEK293T cells were seeded on 60 mm dishes precoated with poly-D-lysine (PDL) and maintained in a humidified incubator at 37℃ with 5% CO2. When the density of cells reached 70% confluent, cells were transfected with plasmids (GFP, Nrxn1β^-^+tdT, Nlgn4+GFP, Nlgn3-EGFP, S-MAG+tdT, and L-MAG+tdT) by Lipo2000 (Invitrogen). 48 hours after transfection, harvest cells for aggregation assay. Cells were washed with warmed PBS twice, added 1ml of warmed 10 mM EDTA in PBS to each well, and incubated at 37℃ for 5 minutes. Then 800 µl cultured medium was added into each well and the cells were detached by tapping on the bottom of the dishes gently. Cells were transferred into new 2 ml tubes, and centrifuged at 1,000 rpm for 5 minutes. The supernatant was removed and resuspend cells were pipetted up and down gently 6 times in 800 µl cultured medium with 10 mM CaCl_2_ and 10 mM MgCl_2_. 200 µl resuspended cells were transferred into a 1.5 ml incubation tube of each condition for a 1:1 mix in a final total volume of 400 µl. 40 µl of each condition was pipetted after mixing onto a charged slice and imaged under a fluorescence microscope as “Time 0”. Tubes were incubated at room temperature in a slow tube rotator for 1 hour and 40 µl each condition was pipetted onto a charged slide and image as “Time 60”.

**Immunoblotting**: Immunoblotting was performed with fluorescently labeled secondary antibodies and Licor detection as described previously [4, 5]. Nlgn3-cKO and their littermate control mice (P24-P28, after tamoxifen injection starting at P14 for 5 days) were deeply anesthetized with isoflurane and the cerebellums were dissected out and lysed with lysis buffer. Insoluble materials were removed by centrifugation, and 20 µg of protein lysate was size-separated on SDS-polyacrylamide gels and transferred onto nitrocellulose membranes following instructions from BioRad. Membranes were blocked in 5% milk prepared in 0.05% TBS-Tween for 1 hour at room temperature, then incubated with primary antibodies overnight at 4°C. Membranes then were washed with 0.05% TBS-Tween (3 times) and then incubated with a fluorescence-labeled secondary antibody (goat anti-rabbit IRDye 680RD, 1:10,000; goat anti-mouse IRDye 680RD, 1:10,000; goat anti-mouse IR Dye 800CW, 1:10,000; goat anti-rabbit IR Dye 800CW, 1:10,000; donkey anti-guinea pig IR Dye 800CW, 1:10,000; donkey anti-guinea pig IR Dye 680RD, 1:10,000; LI-COR Bioscience) were used and signals were detected with an Odyssey Infrared Imager and Odyssey software (LI-COR Biosciences). The total intensity values are calculated by Odyssey software and each value is normalized to actin first and then normalized to control. Antibodies used were as follows: Neuroligin-1 (1:500, mouse monoclonal antibody 129111(4C12), Synaptic System); Neuroligin-2 (1:500, Rabbit polyclonal antibody 129203, Synaptic System); Neuroligin-3 (1:500, mouse monoclonal antibody 129311, Synaptic System); Nrxn1α (1:500, rabbit polyclonal antibody Af870, frontier institute); GLT1 (1:500, rabbit polyclonal antibody 250203, Synaptic System); GluA1 (1:1000, rabbit polyclonal antibody AB1504, Millipore); GluA4 (1:1000, rabbit polyclonal antibody AB1508, Millipore); Synaptophysin (1:1000, rabbit monoclonal antibody ab52636, Abcam); vGluT1(1:1000, Guinea pig polyclonal antibody 135304, Synaptic System); vGluT2(1:1000, rabbit polyclonal antibody 135402, Synaptic System); PSD95 (1:500, mouse monoclonal antibody 124011, Synaptic System); VGAT (1:500, rabbit polyclonal antibody 131002, Synaptic System); Gephyrin (1:500, mouse monoclonal antibody 147111, Synaptic System); GAD67 (1:500, mouse monoclonal antibody MAB5406, Millipore); Calbindin (1:1000, mouse monoclonal antibody C9848, Sigma-Aldrich); Mag (1:1000, mouse monoclonal antibody sc-166849, Sentacruz); β-actin (1:3,000, mouse monoclonal antibody A1978, Sigma-Aldrich).

**qRT-PCR:** Total RNA was isolated and purified from mice at the age of P24-P28 from NL3-cKO and littermate control mice (after tamoxifen injection starting at P14 for 5 days) using Trizol according to the manufacturer’s protocol, and complementary DNA synthesis was performed by reverse transcription of each sample using a TransScript® One-Step gDNA Removal and cDNA Synthesis SuperMix (AT311, Transgen, China). Real-time PCR was performed using the QuantStudio™ 5 Real-Time PCR System(Applied Bio-systems, USA) with SYBR Green detection(AQ131, Transgen, China) in a two-step reaction. ΔC_T_ values were calculated and used to determine the relative mRNA levels of target genes compared to the reference gene. ΔΔC_T_ represented the difference between the ΔC_T_ of the control group and the Nlgn3 condition knockdown group. Data were expressed as 2^-ΔΔCT^. Primer sequences are provided in the Key resources table.

**Immunofluorescence analysis:** Bergman glia plated on coverslips were washed in PBS and then fixed with 4% paraformaldehyde (PFA), permeabilized with 0.3% Triton X-100 in PBS for 10 min, and blocked with 5% BSA/PBS for 1 h at room temperature, and incubated overnight at 4℃in a humidified chamber with primary antibodies anti-S100, 1:1000 (guinea pig polyclonal antibody, 287004, Synaptic System), anti-HA,1:500 (rabbit monoclonal antibody 3727s, cell signaling) followed by PBS wash and incubated with fluorescence-conjugated secondary antibodies (1:1000, Alexa 488, 545, Invitrogen) for 1 h at room temperature. Following another round of PBS washing, the cells were mounted using fluoromount-G (SouthernBiotech, Cat NO 0100-01) and left to dry overnight at room temperature. Coverslips were imaged immediately or stored at 4℃. All coverslips were imaged with a Zeiss LSM980 with a 63x oil objective (PlanApo, NA1.4). For analyzing astrocytes and Bergman glia volume, serial confocal z-stack images (0.2 µm intervals for 2 µm at 1024 × 1024 resolution) were acquired. Images were analyzed in Fiji/Image J and the backgrounds were threshold but kept the same for cortical astrocytes and Bergman glial cells.

**Immunohistochemistry:** Immunohistochemistry experiments and other morphological studies were performed essentially as described previously [4, 5]. Mice at the age of P24-P28 from Nlgn3-cKO and littermate control mice (after tamoxifen injection starting at P14 for 5 days) were anesthetized with isoflurane, and perfused with PBS followed by 4% paraformaldehyde (PFA) in 0.1 M PBS. The dissected cerebellum was post-fixed in 4% PFA overnight at 4°C and cryoprotected in 30% sucrose (in 1× PBS) for 48 h at 4°C. Sagittal brain sections (30 µm) were collected using a cryostat (Leica CM1050) at -20°C. Sections were washed with PBS, blocking with a solution containing 0.3 % Triton X-100 and 5 % goat serum for 30 minutes at room temperature with gentle agitation, and then incubated with primary antibodies overnight at 4 °C (S100, 1:1000, guinea pig polyclonal antibody, 287004, Synaptic System；GLT1, 1:500, rabbit polyclonal antibody 250203, Synaptic System; GluA1,1:1000, rabbit polyclonal antibody AB1504, Millipore; GluA4 ,1:1000, rabbit polyclonal antibody AB1508, Millipore; vGluT1 ,1:1000, Guinea pig polyclonal antibody 135304, Synaptic System; vGluT2, 1:1000, rabbit polyclonal antibody 135402, Synaptic System; VGAT, 1:500, rabbit polyclonal antibody 131002, Synaptic System; Calbindin, 1:1000, mouse monoclonal antibody C9848, Sigma-Aldrich; IBA 1, 1:1000, rabbit polyclonal antibody, 234003, Synaptic System; Parvalbumin, 1:1000, mouse monoclonal antibody P3088, Sigma-Aldrich; NeuN,1:1000, rabbit polyclonal antibody, ABN78, Millipore; HA,1:500, rabbit monoclonal antibody 3727s, cell signaling. Sections were washed 4 times (15 mins each time) in PBS, then treated with secondary antibodies (1:1000, Alexa 488, 545, 633, Invitrogen) for 2 hours at room temperature, and washed 4 times (15 mins each time) again with PBS. Sections were then mounted on superfrost slides (Vectashield, Vector Labs). Serial confocal z-stack images were acquired using a Nikon confocal microscope (A1Rsi) with a 60× oil objective (PlanApo, NA1.4) or Zeiss LSM980 with a 40x oil objective (PlanApo, NA1.3). All acquisition parameters were kept constant among different conditions within experiments. On average, >8 images were collected from cerebellar lobules IV/V (areas of sulci were excluded) from a single animal (N≥3 animals per condition). Images were analyzed in Fiji/Image J and Image backgrounds were threshold but kept the same for all Bergman glia cells both in control and Aldh1L1Cre-Nlgn3 mice. For each experiment >25 images per condition were collected and analyzed.

**Electron microscopy:** Electron microscopy experiments were carried out as described previously [5]. Mice at the age of P24-P28 from Nlgn3-cKO and littermate control mice (after tamoxifen injection starting at P14 for 5 days) were deeply anesthetized and transcardially perfused with 0.01M PBS (pH7.4) followed by 4% paraformaldehyde (PFA) with 0.075% glutaraldehyde in 0.1 M PBS (pH7.4). The dissected cerebellum was post-fixed in the fixative solution containing 4% glutaraldehyde in phosphate buffer with 8% sucrose overnight at 4°C. 200-300 µm sagittal cerebellar sections were post-fixed with 1% OsO_4_, dehydrated in a graded series of ethanol, and embedded in Epon812. All cerebellar samples were trimmed to focus only on lobules IV/V of the cerebellar cortex. Ultrathin sections (70 nm) were cut using an ultramicrotome (Reichert-Nissei Ultracut S; Lecia, Austria) and then mounted on single-slot grids coated with a pioloform membrane (Agar Scientific, Stansted, UK), stained with an aqueous solution of 2% uranyl acetate followed by 1% lead citrate. Images from the upper third of the molecular layer were taken at 20,000x magnification. Since the majority of excitatory synapses in the upper third of the molecular layer are from PF-PC synapses excitatory synapses were readily identified with postsynaptic density. Therefore, the density of PSD is used to analyze the density of the PF-PC synapse. Per genotype 2 animals were processed and about 20 random images/mice were taken with the electron microscope (JEM-1400; JEOL; Tokyo, Japan). For imaging and analysis, the experimenter was blinded for all images that were studied. PF-PC synapse density was counted using ImageJ.

**Electrophysiology**: Electrophysiological experiments were carried out as described previously [4, 5]. Mice of NL3-cKO and control littermate (aged P24-P28, after tamoxifen injection starting at P14 for 5 days) were deeply anesthetized with isoflurane and decapitated. Brains were rapidly transferred into ice-cold low-Ca^2+^ artificial CSF (aCSF). (aCSF) containing (in mM): 125 NaCl, 2.5 KCl, 3 MgCl_2_, 0.1 CaCl_2_, 25 glucose, 1.25 NaH_2_PO_4_, 0.4 ascorbic acid, 3 myo-inositol, 2 Na-pyruvate, and 25 NaHCO_3_; pH was adjusted to 7.4 by continuous gassing with carbogen. Coronal sections (250 µm) containing layer II/III of the somatosensory cortex and Sagittal slices of 250 µm cerebellum were obtained using a vibratome (VT 1200S; Leica) and kept at room temperature (22-25°C) for >1 hour before recordings, then were transferred to a recording chamber and were perfused with oxygenated aCSF containing (in mM): 1 MgCl_2_, 2 CaCl_2_ instead of 3 MgCl_2_, 0.1 CaCl_2_. Whole-cell recordings from Purkinje cells (PCs) in cerebellar lobules IV/V (voltage-clamped at -70 mV for PC) were performed at room temperature with borosilicate glass pipettes (3-4 MΩ for PCs) pulled with vertical micropipette puller (PC-10, Narishige). The internal pipette solutions contained (in mM) the following components for the following recordings: Voltage-clamp EPSC recordings from PCs, 140 Cs-gluconate, 5 CsCl, 2 MgCl_2_, 0.5 EGTA, 2 Na-ATP, 0.5 NaGTP (pH 7.3, adjusted with CsOH); or all EPSC recordings, the aCSF bath solution contained 50 µM picrotoxin, 10 µM APV; climbing fiber EPSC recordings additionally included 0.5 µM NBQX to reduce the EPSC size and minimize the clamping error. Focal square pulse stimuli (duration 50 µs, amplitude 0-30 V or 1-100 µA) were applied with a bipolar stimulation electrode (FHC, Bowdoinham, ME). Climbing fibers EPSCs were acquired by stimulation in the granule cell layer near Purkinje cells and identified by their characteristic all-or-none response and paired-pulse depression at a 50 ms inter-stimulus interval. Intensive mapping with stimulus electrodes is performed to determine the multiple climbing fibers innervation of the Purkinje cell [6]. When recording PF-PC EPSCs, the stimulator was put in the distal molecular layer (~200 µm from the recorded Purkinje cell). The PF-PC EPSCs were identified by their characteristic paired-pulse facilitation at 50 ms intervals. Miniature ESPC (mEPSC) recordings were performed with 1 µM TTX in the aCSF. The majority (>95%) of mEPSC events are from parallel fibers, because of mEPSC from parallel fibers with decay time >1 ms and mEPSC from climbing fibers with decay time <1 ms [7–9]. Only mEPSC with decay time > 1 ms were included for further analysis. NBQX (20 µm) was used to block AMPAR-mediated current. Only those recordings of PCs with a stable series resistance (8-9 MΩ) were used for further analysis. The recordings from the pyramidal neurons in the somatosensory cortex were performed with a drug cocktail (1 µM TTX + 50 µm picrotoxin + 50 µm APV) for mEPSC or a drug cocktail (1 µM TTX + 20 µm NBQX and 50 µm APV) for mIPSC in the aCSF. The series resistance (measured by applying 10 ms, -10 mV voltage pulses) was not compensated and monitored before and after experiments.

**Behavioral tests:** Behavioral tests were conducted during the day (light-on period) using age-matched male littermates (2-3 months) from 10 breeding cages. All mice from 1 cohort were handled by the operator for 2-3 minutes per day for 5 consecutive days for habituation. Behaviors were tested in the following order: open field exploration, three-chamber test, and rotarod test. Rest for at least 1 day between different tests.

**Open field test:** The open field test was performed in a square gray plastic arena (45 cm length x 45 cm width x 45 cm height), and the center zone line was 10 cm from the edge. Mice were placed in the center of the chamber and their movements were recorded for 30 minutes with a video camera, the surface of the arena was cleaned with 70% ethanol between trials. Moved distance spent in the central area and in the periphery of the arena were analyzed using Viewer 3.0.

**Rotarod test:** Rotarod testing consists of 3 trials per day over 4 consecutive days (Trial 1-12). Program the equipment as starting from 4-40 rpm over 5 min (trial 1-6) and 8-80 rpm (trial 7-12) within 5 min and the latency to fall was recorded for each trial. 15 min interval between each trial. Each trial ended when a mouse fell off, made one complete backward revolution while hanging on, or reached 300s.

**Three-chamber social interaction test:** The three-chambered apparatus was 60 cm [L] x 40 cm [W] x 25 cm [H] with a 20-cm-wide center chamber and 20-cm-wide side chambers. Testing occurs in three sessions: habituation, sociability, and social novelty. In the habituation session, Astro-Nlgn3-KO or littermate control mice were placed into the center chamber and allowed to explore both the left and right chambers for 10 minutes. In the sociability session, an age-matched stranger was placed in one side chamber, the test animal was allowed to explore the social apparatus for 10 minutes freely. Subsequently, in the social novelty session, another age-matched stranger was placed in the other empty side chamber, and the test animal was allowed to freely explore the social apparatus for another 10 minutes. The box was wiped with 70% ethanol and air-dried between mice. Behaviors were recorded using a video camera, and the sniffing time was manually and blindly calculated by independent observers. Sniffing time was defined as instances where the mouse's nose contacted the side chamber or when the mouse exhibited orientation towards the side chamber within a distance of 2 cm. Additionally, the difference index was calculated, which was the numerical difference between the times spent exploring the targets (Stranger 1 versus Empty and Stranger 2 versus Stranger 1) divided by the total time spent exploring both targets.

**Table S1 The number of cells in different clusters**

|  |  | **all** | **Astro-NIgn3-KO** | **Ctrl** |
| --- | --- | --- | --- | --- |
|  | **all** | 46654 | 23497 | 23157 |
|  |  |  |  |  |
| **Unsupervised** | DCN Oligodendrocyte | 5384 | 2539 | 2845 |
|  | WM Oligodendrocyte | 8592 | 4197 | 4395 |
|  | Molecular layer IN | 9053 | 4548 | 4505 |
|  | Bergmann glia | 2928 | 1472 | 1456 |
|  | Purkinje cell | 4900 | 2411 | 2489 |
|  | Granule cell | 15797 | 8330 | 7467 |
|  |  |  |  |  |
|  |  |  |  |  |
| **Unsupervised+prior knowledge** | DCN Oligodendrocyte | 5384 | 2539 | 2845 |
|  | WM Oligodendrocyte | 8592 | 4197 | 4395 |
|  | Molecular layer IN | 9053 | 4548 | 4505 |
|  | Bergmann glia | 2565 | 1272 | 1293 |
|  | Purkinje cell | 4320 | 2114 | 2206 |
|  | Granule cell | 12747 | 6485 | 6262 |
|  | “filtered” Purkinje cell | 580 | 297 | 283 |
|  | “filtered” Bergmann glia | 363 | 200 | 163 |
|  | “filtered” Granule cell | 3050 | 1845 | 1205 |

**Table S2 Differentially expressed genes of unsupervised clustering**

| **p_val** | **avg_log2FC** | **pct.1** | **pct.2** | **p_val_adj** | **cluster** | **gene** |
| --- | --- | --- | --- | --- | --- | --- |
| 4.47E-21 | -0.27596 | 0.957 | 0.935 | 9.10E-18 | Granule cell | Mbp |
| 1.58E-07 | -0.25887 | 0.814 | 0.77 | 0.000322 | Granule cell | Chn2 |
| 2.75E-20 | -0.25949 | 0.901 | 0.676 | 5.60E-17 | Purkinje cell | Trf |
| 5.34E-17 | -0.25713 | 0.826 | 0.536 | 1.09E-13 | Purkinje cell | Fabp7 |
| 1.61E-06 | -0.40977 | 0.982 | 0.96 | 0.003276 | Purkinje cell | Aldoc |
| 2.44E-05 | -0.28422 | 0.93 | 0.902 | 0.049797 | Purkinje cell | Slc1a3 |
| 5.69E-28 | -0.34576 | 0.953 | 0.683 | 1.16E-24 | Bergmann glia | Enpp2 |
| 2.95E-14 | -0.27878 | 0.95 | 0.935 | 6.01E-11 | Bergmann glia | Fth1 |
| 7.06E-13 | -0.34692 | 0.951 | 0.935 | 1.44E-09 | Bergmann glia | Mbp |
| 4.32E-12 | -0.36158 | 0.984 | 0.98 | 8.80E-09 | Bergmann glia | Pcp2 |
| 4.51E-12 | -0.31288 | 0.982 | 0.981 | 9.20E-09 | Bergmann glia | Pet100 |
| 4.22E-10 | -0.2755 | 0.908 | 0.667 | 8.59E-07 | Bergmann glia | Mag |
| 1.02E-05 | -0.40365 | 0.937 | 0.864 | 0.020714 | Bergmann glia | Ttr |
| 1.12E-37 | -0.2755 | 0.897 | 0.885 | 2.29E-34 | Molecular layer IN | Fth1 |
| 9.39E-34 | -0.34533 | 0.944 | 0.927 | 1.91E-30 | Molecular layer IN | Mbp |
| 1.65E-14 | -0.32205 | 0.933 | 0.883 | 3.36E-11 | Molecular layer IN | Pet100 |
| 4.39E-14 | -0.34429 | 0.94 | 0.892 | 8.95E-11 | Molecular layer IN | Pcp2 |
| 5.05E-13 | -0.33225 | 0.692 | 0.619 | 1.03E-09 | Molecular layer IN | Mobp |
| 1.80E-12 | -0.34402 | 0.947 | 0.902 | 3.67E-09 | Molecular layer IN | Igsf5 |
| 2.06E-10 | -0.27753 | 0.76 | 0.529 | 4.19E-07 | Molecular layer IN | Nefl |
| 1.66E-06 | -0.27633 | 0.906 | 0.842 | 0.003376 | Molecular layer IN | Ttr |
| 1.70E-07 | -0.26161 | 0.905 | 0.875 | 0.000347 | WM Oligodendrocyte | Aldoc |
| 5.64E-06 | -0.32118 | 0.863 | 0.677 | 0.011491 | WM Oligodendrocyte | Nefl |
| 3.35E-41 | 0.306342 | 0.763 | 0.515 | 6.82E-38 | DCN Oligodendrocyte | Kcna1 |
| 1.57E-15 | -0.42259 | 0.965 | 0.723 | 3.19E-12 | DCN Oligodendrocyte | Ccdc153 |
| 1.62E-10 | -0.28394 | 0.918 | 0.644 | 3.30E-07 | DCN Oligodendrocyte | Tmem212 |
| 5.43E-07 | -0.33939 | 0.827 | 0.62 | 0.001106 | DCN Oligodendrocyte | Lrrc61 |
| 2.06E-05 | -0.32423 | 0.855 | 0.7 | 0.041986 | DCN Oligodendrocyte | Igf2 |

**Table S3 Differentially expressed genes of unsupervised clustering + prior knowledge**

| **p_val** | **avg_log2FC** | **pct.1** | **pct.2** | **p_val_adj** | **cluster** | **gene** |
| --- | --- | --- | --- | --- | --- | --- |
| 1.60E-07 | -0.29896 | 0.835 | 0.509 | 0.000325 | “filtered” Bergmann glia | Fgf2 |
| 1.07E-06 | -1.06502 | 0.97 | 0.571 | 0.00217 | “filtered” Bergmann glia | Map7d3 |
| 2.06E-09 | -0.33248 | 0.835 | 0.558 | 4.19E-06 | “filtered” Purkinje cell | Nnat |

**Table S4 Differentially expressed genes of unsupervised clustering in V1 cortex**

| **avg_log2FC** | **pct.1** | **pct.2** | **p_val_adj** | **cluster** | **gene** |
| --- | --- | --- | --- | --- | --- |
| 0.367048 | 0.446 | 0.951 | 5.91E-43 | Sst-IN | Myl4 |
| 0.323491 | 0.456 | 0.936 | 7.76E-36 | Sst-IN | Phax |
| -0.29338 | 0.079 | 0.336 | 5.10E-32 | Sst-IN | Agbl2 |
| 0.359044 | 0.301 | 0.825 | 2.07E-30 | Sst-IN | C1ql2 |
| 0.253642 | 0.412 | 0.881 | 1.34E-21 | Sst-IN | Dmac1 |
| 0.306159 | 0.419 | 0.834 | 7.45E-18 | Sst-IN | Ppil4 |
| -0.26106 | 0.552 | 0.874 | 2.54E-11 | Sst-IN | Dlg2 |
| -0.50845 | 0.239 | 0.67 | 2.59E-10 | Sst-IN | Vac14 |
| -0.312 | 0.586 | 0.932 | 5.55E-10 | Sst-IN | Cnp |
| -0.42039 | 0.666 | 0.975 | 1.74E-06 | Sst-IN | Mobp |
| 0.254003 | 0.206 | 0.619 | 2.52E-06 | Sst-IN | Mgp |
| -0.45445 | 0.078 | 0.388 | 4.49E-06 | Sst-IN | Gpr39 |
| -0.29103 | 0.245 | 0.45 | 0.000165 | Sst-IN | Gfm2 |
| -0.27588 | 0.909 | 0.967 | 0.000858 | Sst-IN | Mbp |
| -0.28784 | 0.999 | 1 | 0.005707 | Sst-IN | Fth1 |
| -0.30305 | 0.344 | 0.849 | 2.49E-36 | Pvalb-IN | Gtpbp4 |
| 0.331788 | 0.322 | 0.889 | 7.92E-32 | Pvalb-IN | Spink8 |
| 0.335091 | 0.401 | 0.882 | 1.84E-26 | Pvalb-IN | Tcap |
| 0.305437 | 0.431 | 0.873 | 3.14E-20 | Pvalb-IN | Rpp25l |
| 0.356431 | 0.596 | 0.959 | 2.39E-13 | Pvalb-IN | Myl4 |
| 0.33369 | 0.357 | 0.81 | 3.44E-13 | Pvalb-IN | Adat2 |
| -0.27563 | 0.526 | 0.853 | 3.94E-12 | Pvalb-IN | Vip |
| 0.37213 | 0.389 | 0.822 | 3.08E-11 | Pvalb-IN | C1ql2 |
| 0.284037 | 0.446 | 0.873 | 1.38E-10 | Pvalb-IN | Pdyn |
| 0.318747 | 0.628 | 0.966 | 3.57E-10 | Pvalb-IN | Calb1 |
| 0.319786 | 0.424 | 0.389 | 1.39E-09 | Pvalb-IN | Tshz2 |
| 0.268027 | 0.382 | 0.755 | 3.41E-06 | Pvalb-IN | Dnal4 |
| 0.326273 | 0.436 | 0.791 | 5.28E-06 | Pvalb-IN | Thap11 |
| 0.383458 | 0.544 | 0.904 | 5.86E-06 | Pvalb-IN | Zmynd11 |
| 0.49984 | 0.616 | 0.969 | 2.12E-05 | Pvalb-IN | Nhp2 |
| 0.309387 | 0.504 | 0.832 | 2.27E-05 | Pvalb-IN | Rhno1 |
| 0.310132 | 0.254 | 0.637 | 2.47E-05 | Pvalb-IN | Mgp |
| -0.27474 | 0.509 | 0.769 | 0.000281 | Pvalb-IN | Pias4 |
| 0.281947 | 0.434 | 0.755 | 0.001228 | Pvalb-IN | Ube2f |
| 0.286171 | 0.546 | 0.93 | 0.002812 | Pvalb-IN | Mal |
| 0.281736 | 0.359 | 0.74 | 0.005116 | Pvalb-IN | Sat2 |
| 0.454077 | 0.332 | 0.678 | 0.007369 | Pvalb-IN | Cdc42ep3 |
| 0.260333 | 0.437 | 0.924 | 1.91E-73 | Cck-IN | Rasl10a |
| 0.302326 | 0.441 | 0.928 | 6.92E-70 | Cck-IN | H2-DMa |
| -0.31584 | 0.143 | 0.343 | 2.73E-42 | Cck-IN | Gpr39 |
| -0.31957 | 0.512 | 0.911 | 3.96E-28 | Cck-IN | Mobp |
| 0.343799 | 0.589 | 0.966 | 6.38E-25 | Cck-IN | Gadd45g |
| 0.350183 | 0.556 | 0.993 | 5.59E-23 | Cck-IN | Tesc |
| 0.258663 | 0.432 | 0.787 | 8.95E-23 | Cck-IN | Taok3 |
| 0.272328 | 0.569 | 0.961 | 1.70E-21 | Cck-IN | Ak5 |
| -0.31366 | 0.801 | 0.964 | 7.68E-11 | Cck-IN | Plp1 |
| -0.3231 | 0.781 | 0.947 | 1.27E-10 | Cck-IN | Mbp |
| 0.297292 | 0.612 | 0.93 | 6.10E-09 | Cck-IN | Calb1 |
| 0.342102 | 0.379 | 0.714 | 3.77E-07 | Cck-IN | Snrpb2 |
| 0.457682 | 0.739 | 1 | 4.40E-05 | Cck-IN | Lypd1 |
| 0.275901 | 0.453 | 0.931 | ####### | Excitatory neuron | Myl4 |
| 0.261042 | 0.495 | 0.974 | 4.98E-96 | Excitatory neuron | Rasl10a |
| -0.25265 | 0.146 | 0.345 | 2.34E-58 | Excitatory neuron | Gpr39 |
| 0.321056 | 0.632 | 0.987 | 2.48E-29 | Excitatory neuron | Crym |
| -0.27127 | 0.317 | 0.46 | 4.46E-19 | Excitatory neuron | Gfm2 |
| 0.250543 | 0.568 | 0.844 | 8.34E-05 | Excitatory neuron | Flywch1 |
| -0.26795 | 0.321 | 0.492 | 0.030662 | Excitatory neuron | Col19a1 |
| 0.326695 | 0.451 | 0.922 | 1.48E-28 | Astrocyte | Srrd |
| 0.433126 | 0.582 | 0.971 | 1.79E-28 | Astrocyte | Cplx3 |
| 0.3114 | 0.488 | 0.953 | 2.76E-24 | Astrocyte | Nhp2 |
| -0.3126 | 0.367 | 0.794 | 2.63E-15 | Astrocyte | Dnajc24 |
| 0.276367 | 0.589 | 0.946 | 1.99E-14 | Astrocyte | Rasl10a |
| 0.253206 | 0.545 | 0.961 | 1.68E-13 | Astrocyte | Cort |
| -0.32174 | 0.37 | 0.778 | 2.03E-12 | Astrocyte | Hes5 |
| 0.254743 | 0.384 | 0.772 | 9.23E-12 | Astrocyte | Rhno1 |
| 0.276542 | 0.414 | 0.821 | 1.29E-08 | Astrocyte | Lrrtm4 |
| -0.25304 | 0.384 | 0.792 | 3.36E-08 | Astrocyte | Aebp1 |
| 0.26591 | 0.461 | 0.79 | 6.48E-06 | Astrocyte | Tmem117 |
| -0.2609 | 0.485 | 0.798 | 0.000244 | Astrocyte | Tln1 |
| 0.648857 | 0.566 | 0.874 | 0.00062 | Astrocyte | Phax |
| 0.269103 | 0.508 | 0.772 | 0.005339 | Astrocyte | Cox6a2 |
| 0.472399 | 0.532 | 0.887 | 0.014449 | Astrocyte | Nkx6-2 |
| 0.361452 | 0.431 | 0.971 | 6.33E-35 | Astrocyte/Oligodendrocyte | Cplx3 |
| 0.31579 | 0.353 | 0.832 | 7.33E-20 | Astrocyte/Oligodendrocyte | Mrpl35 |
| 0.264329 | 0.274 | 0.779 | 8.62E-18 | Astrocyte/Oligodendrocyte | Tmem126b |
| -0.32066 | 0.231 | 0.355 | 2.41E-16 | Astrocyte/Oligodendrocyte | Zfp804b |
| 0.256702 | 0.266 | 0.744 | 3.94E-14 | Astrocyte/Oligodendrocyte | Myo5b |
| 0.284192 | 0.416 | 0.88 | 1.79E-13 | Astrocyte/Oligodendrocyte | Wipi1 |
| 0.287954 | 0.224 | 0.717 | 8.90E-13 | Astrocyte/Oligodendrocyte | Tfam |
| -0.25046 | 0.421 | 0.861 | 2.60E-12 | Astrocyte/Oligodendrocyte | Magi2 |
| 0.359534 | 0.422 | 0.829 | 7.83E-09 | Astrocyte/Oligodendrocyte | Sdhaf2 |
| 0.27137 | 0.348 | 0.792 | 1.87E-08 | Astrocyte/Oligodendrocyte | Dtnbp1 |
| -0.29196 | 0.414 | 0.779 | 1.27E-05 | Astrocyte/Oligodendrocyte | Sema7a |
| -0.34687 | 0.274 | 0.424 | 7.41E-05 | Astrocyte/Oligodendrocyte | Plcxd3 |
| -0.27617 | 0.366 | 0.741 | 8.20E-05 | Astrocyte/Oligodendrocyte | Scamp2 |
| 0.327703 | 0.471 | 0.96 | 0.001378 | Astrocyte/Oligodendrocyte | Npy |
| 0.294925 | 0.619 | 0.981 | 0.047296 | Astrocyte/Oligodendrocyte | Enc1 |

**Resources table**

| Reagent or resources | Source | Identifier |
| --- | --- | --- |
| **Antibodies** |  |  |
| Mouse anti-Neuroligin 3 | Synaptic Systems | Cat#129311;RRID:AB_2151947 |
| Rabbit anti-Neuroligin 2 | Synaptic Systems | Cat# 129203;RRID:AB_993014 |
| Mouse anti-Neuroligin 1 | Synaptic Systems | Cat# 129111;RRID: AB_887747 |
| Rabbit anti-Nrxn1α | frontier institute | Cat#Af870 |
| Rabbit anti-EAAT 2 | Synaptic Systems | Cat# 250203; RRID: AB_11042312 |
| Rabbit anti-GluA1 | Millipore | Cat#ab1504;RRID: AB_2113602 |
| Rabbit anti-GluA4 | Millipore | Cat#AB1508;RRID: AB_90711 |
| Guinea pig anti-VGluT1 | Synaptic Systems | Cat#135304; RRID: AB_887878 |
| Rabbit anti-vGluT2 | Synaptic Systems | Cat# 135402;RRID: AB_2187539 |
| Mouse anti-PSD95 | Synaptic Systems | Cat# 124011;RRID: AB_10804286 |
| Guinea pig anti-vGAT | Synaptic Systems | Cat#131004; RRID: AB_887873 |
| Rabbit anti-vGAT | Synaptic Systems | Cat#131002; RRID: AB_887871 |
| Mouse anti-GAD67 | Millipore | Cat#MAB5406;RRID: AB_2278725 |
| Mouse anti-Gephyrin | Synaptic Systems | Cat# 147111;RRID: AB_887719 |
| Rabbit anti-Synaptophysin | Abcam | Cat# ab52636;RRID: AB_882786 |
| Mouse anti-Calbindin | Sigma-Aldrich | Cat# C9848;RRID: AB_476894 |
| Guinea pig anti-S100B | Synaptic Systems | Cat# 287004;RRID: AB_2620025 |
| Rabbit anti-IBA 1 | Synaptic Systems | Cat# 234003;RRID: AB_10641962 |
| Mouse anti-Parvalbumin | Sigma P3088 | Cat#P3088; RRID: AB_477329 |
| Rabbit anti-NeuN | Millipore | Cat# ABN78;RRID: AB_10807945 |
| Mouse anti-β-actin | Sigma-Aldrich | Cat# A1978;RRID: AB_476692 |
| Rabbit anti-HA | Cell signaling | Cat#3724s; RRID: AB_1549585 |
| Mouse anti-Mag | Santacruz | Cat#sc-166849; RRID: AB_2250078 |
| Rabbit anti-GPR78 | Abcam | Cat# ab21685; |
| Mouse anti-Tomm20 | Abcam | Cat# ab56783; RRID: AB_945896 |
| Mouse anti-GM130 | BD Bioscience | Cat# 610823; RRID: AB_398142 |
| Rabbit anti Caspase-3 (Asp175) | Cell signaling | Cat# 9961S; RRID: AB_2341188 |
| IRDye 680RD Goat anti-Mouse IgG antibody | LI-COR Biosciences | Cat#926-68070; RRID: AB_10956588 |
| IRDye 680RD Goat anti-Rabbit IgG antibody | LI-COR Biosciences | Cat#926-68071; RRID: AB_10956166 |
| IRDye 800CW Goat anti-Mouse IgG antibody | LI-COR Biosciences | Cat#926-32210; RRID: AB_621842 |
| IRDye 800CW Goat anti-Rabbit IgG antibody | LI-COR Biosciences | Cat#926-32211; RRID: AB_621843 |
| IRDye 800CW Donkey anti-Guinea Pig IgG antibody | LI-COR Biosciences | Cat# 926-32411; RRID: AB_1850024 |
| IRDye 680RD Donkey anti-Guinea Pig IgG antibody | LI-COR Biosciences | Cat#926-68077; RRID: AB_10956079 |
| Goat anti-Mouse IgG (H+L) Highly Cross-adsorbed Secondary antibody, Alexa Flour 633 | Thermo Fisher Scientific | Cat#A21052; RRID: AB_2535719 |
| Goat anti-Guinea Pig IgG (H+L) Highly Cross-adsorbed Secondary antibody, Alexa Flour 488 | Thermo Fisher Scientific | Cat#A11073; RRID: AB_2534117 |
| Goat anti-Rabbit IgG (H+L) Highly Cross-adsorbed Secondary antibody, Alexa Flour 546 | Thermo Fisher Scientific | Cat#A11035; RRID: AB_2534093 |
| Goat anti-Mouse IgG (H+L) Highly Cross-adsorbed Secondary antibody, Alexa Flour 546 | Thermo Fisher Scientific | Cat#A11030; RRID: AB_2534089 |
| Goat anti-Rabbit IgG (H+L) Highly Cross-adsorbed Secondary antibody, Alexa Flour 488 | Thermo Fisher Scientific | Cat#A11034; RRID: AB_2576217 |
| Goat anti-Guinea Pig IgG (H+L) Highly Cross-adsorbed Secondary antibody, Alexa Flour 568 | Thermo Fisher Scientific | Cat#A11075; RRID: AB_2534119 |
| Goat anti-Guinea Pig IgG (H+L) Highly Cross-adsorbed Secondary antibody, Alexa Flour 647 | Thermo Fisher Scientific | Cat#A21450; RRID: AB_2735091 |
| **Chemicals** |  |  |
| Tetrodotocin citrate (TTX, Na channel blocker) | Calbiochem | Cat No: 554412 |
| NBQX disodium salt | Tocris bioscience | Cat No.1044 |
| CNQX disodium salt | Tocris bioscience | Cat No. 1045 |
| D-AP5 | Tocris Bioscience | Cat No. 0106 |
| Tamoxifen | Sigma-Aldrich | Cat No. T5648 |
| **Experimental Models: Animals** |  |  |
| Aldh1L1.cre/ERT2 | Jackson Laboratory | Stock No. 029655 |
| Mouse: GLAST-CreER | Jackson Laboratory | Stock No. 012586 |
| Nlgn3^fl/+^ | Jackson Laboratory | Stock No. 023398 |
| Mouse: Ai14 | Jackson Laboratory | Stock No. 007908 |
| Mouse: Cas9^fl/+^ mice | Jackson Laboratory | Stock No. 024857 |
| **Software** |  |  |
| Fiji | Imagej.net(USA) | https://imagej.net/software/fiji/ |
| Clampfit 10.7 | Molecular Devices, CA, USA | N/A |
| Igor Pro6.37 | WaveMetrics, Inc. | https://www.wavemetrics.com/software/igor-pro-637-installer-windows-64-bit |
| MATLAB R2020a | MathWorks | https://www.mathworks.com/ |
| Viewer 3 | BIOBSERVE | http://www.biobserve.com/behavioralresearch/products/viewer/ |
| Imaris 10.0 | Oxford Instruments | Order-ID: 9puq-sb95-uarg-gyd3 |
| **Oligonucleotides** |  |  |
| Mouse Mag-F：AAGTGGAATCAGGAGACA | This paper | N/A |
| Mouse Mag-R: GTGGCGAGGAATATCATC | This paper | N/A |
| Mouse MBP-F: ACACGAGAACTACCCATTATGGC | This paper | N/A |
| Mouse MBP-R: CCAGCTAAATCTGCTGAGGGA | This paper | N/A |
| Mouse Opain-F: CACTGAACTTTACACTGCCATCG | This paper | N/A |
| Mouse Opain-R: TACAAGCCACCAAAGTCCTCTTC | This paper | N/A |
| Moues Trf-F: GCTGTCCCTGACAAAACGGT | This paper | N/A |
| Mouse Trf-R: CGGAAGGACGGTCTTCATGTG | This paper | N/A |
| Mouse Fth1-F: CAAGTGCGCCAGAACTACCA | This paper | N/A |
| Mouse Fth1-R: GCCACATCATCTCGGTCAAAA | This paper | N/A |
| Mouse GAPDH-F: AGGTCGGTGTGAACGGATTTG | This paper | N/A |
| Mouse GAPDH-R: TGTAGACCATGTAGTTGAGGTCA | This paper | N/A |
| **Bacterial and virus strains** |  |  |
| pX330 | Dr. Huaqiang Fang | N/A |
| pMiniT vector | Dr. Huaqiang Fang | N/A |
| pcDNA3.1-EGFP | This paper | N/A |
| Nrxn1β^-^ | Gifted from Thomas C. Südhof | N/A |
| hNgln4-WT-HA in FUW | Gifted from Thomas C. Südhof | N/A |
| pcDNA3.1-Nlgn3-EGFP | This paper | N/A |
| pcDNA3.1-L-MAG-flag | This paper | N/A |
| pcDNA3.1-S-MAG-flag | This paper | N/A |
| AAV2/9-HA-Nlgn3-knock-in | This paper | N/A |
| AAV5-CAG-EGFP | Shanghai Genechem Co., LTD | N/A |

**Supplemental Figure and Figure Legends**


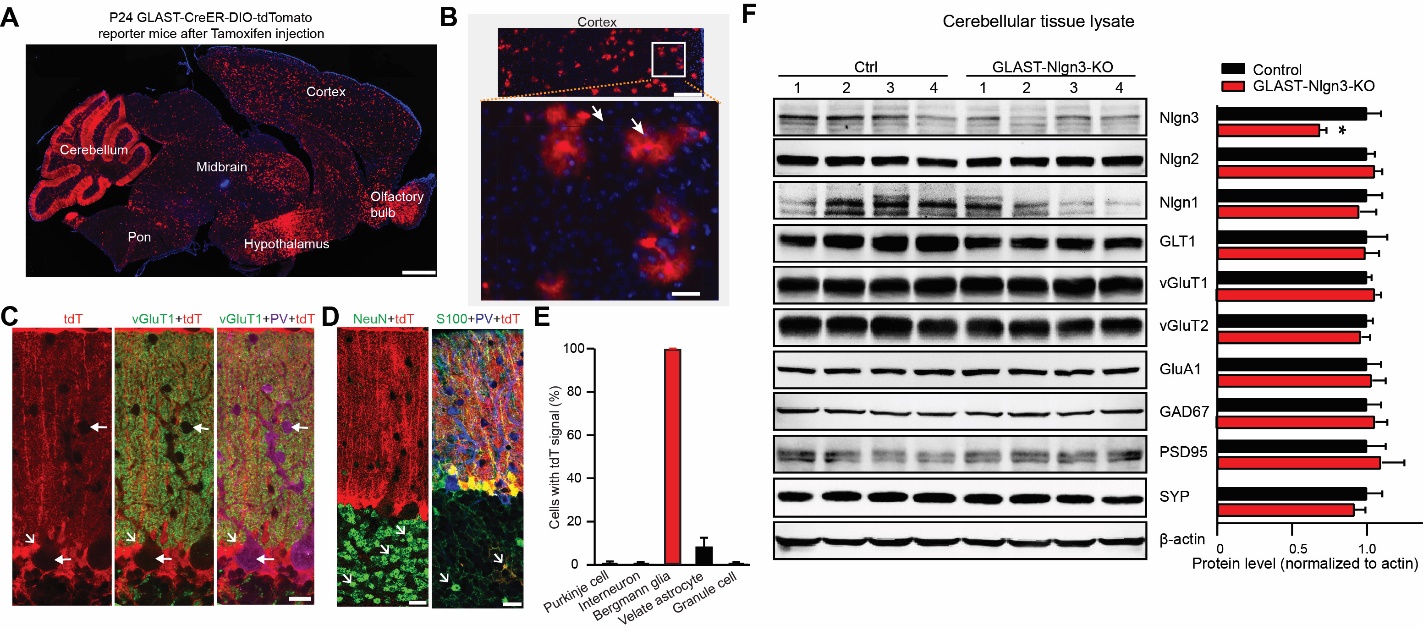


**Figure S1: Western blots of protein prepared from the cerebellum of the P24-P28 treated mice.** (**A**) Representative images of the sagittal section for Glast-CreER-DOI-tdTomato reporter mice after Tamoxifen injection. Animals were injected with tamoxifen (Tam) between P14 and P18 and further staining with cell-specific markers was performed between P24 and P28. (**B**) Representative images of cortical astrocytes in the Glast-CreER-DOI-tdTomato reporter mice. Cre-recombination is sparsely but broadly expressed in cortical astrocytes. (**C**-**D**) Representative images of cerebellum astrocytes with the Glast-CreER-DOI-tdTomato reporter mice. The slices are co-labeled with vGluT1 (green) and PV (blue) or co-stained with S100 (green), PV (blue), and NeuN (green). (**E**) Quantification of co-staining ratio for the tdT with cell-specific markers. (**F**) Western blots of synaptic proteins (Left) and the summary graphs (right) with cerebellum lysis from P24-P28 mice; All data presented as means ± SEM; *P<0.05 by Welch's *t*-test. (n=4 littermate pairs).


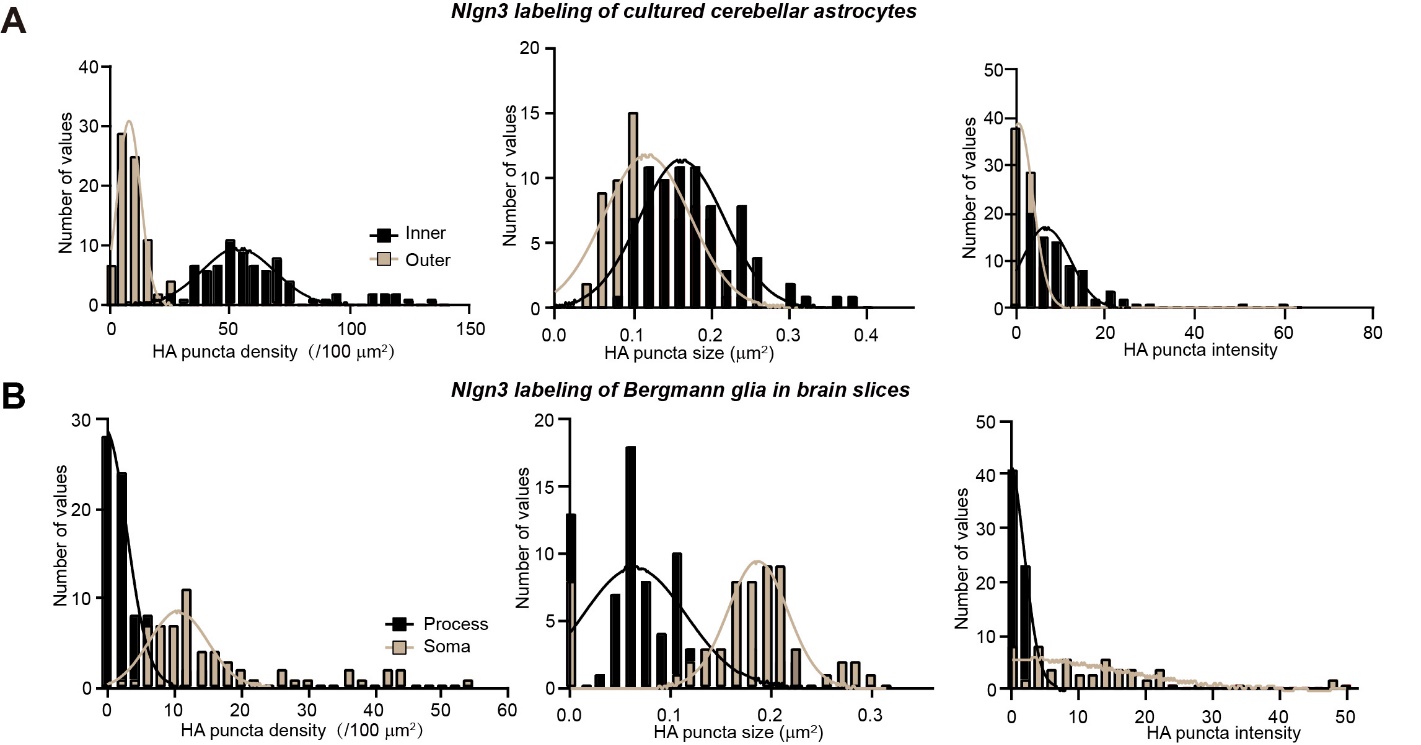


**Figure S2: Detailed analysis of Nlgn3 distribution in cerebellar astrocytes.** (**A**) Histogram distribution of the HA puncta density, puncta size, and puncta intensity in cultured cerebellar astrocytes. (**B**) Histogram distribution of the HA puncta density, puncta size, and puncta intensity in Bergmann glia in brain slices.

**
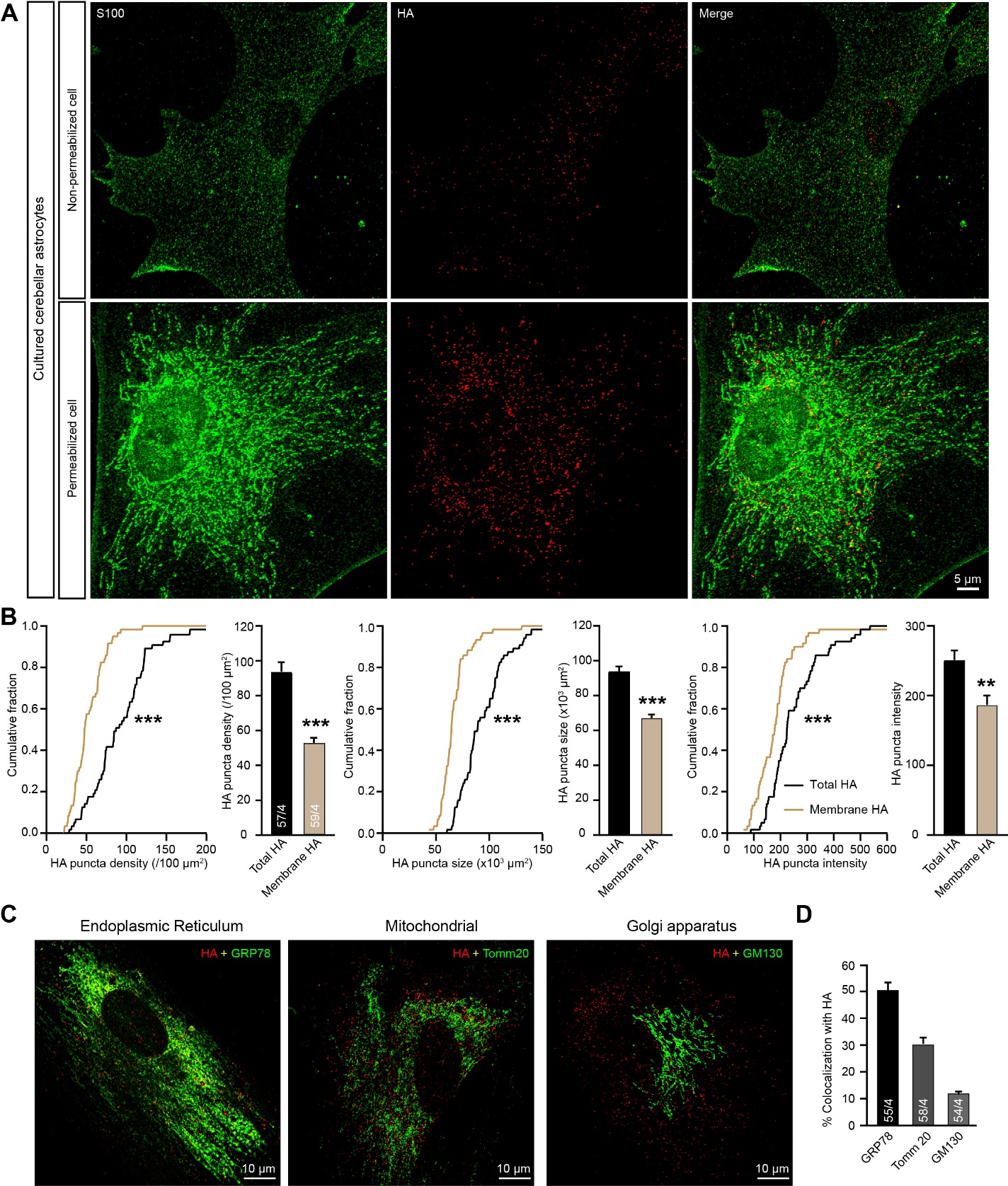
 Figure S3: The subcellular distribution of endogenous Nlgn3 in astrocytes.** (**A**) Representative images showing immunostaining of HA (red) with S100 (green) in permeabilized and non-permeabilized cerebellar astrocytes. (**B**) Cumulative and summary data for the HA puncta density, puncta size, and puncta intensity from (A). (**C**) Representative images showing co-staining of the HA (red) with the endoplasmic reticulum, mitochondrial, and Golgi apparatus markers (green). (**D**) Quantification of the co-staining ratio of the HA (red) with the endoplasmic reticulum, mitochondrial, and Golgi apparatus markers (green) from (C). All data was presented as means ± SEM; Statistical analysis was performed by Welch's *t*-test (bar diagrams) or Kolmogorov-Smirnov test (cumulative distributions); **P<0.01; ***P<0.001. The numbers of cells/independent cultures (B, D) analyzed are shown in the bar graphs.


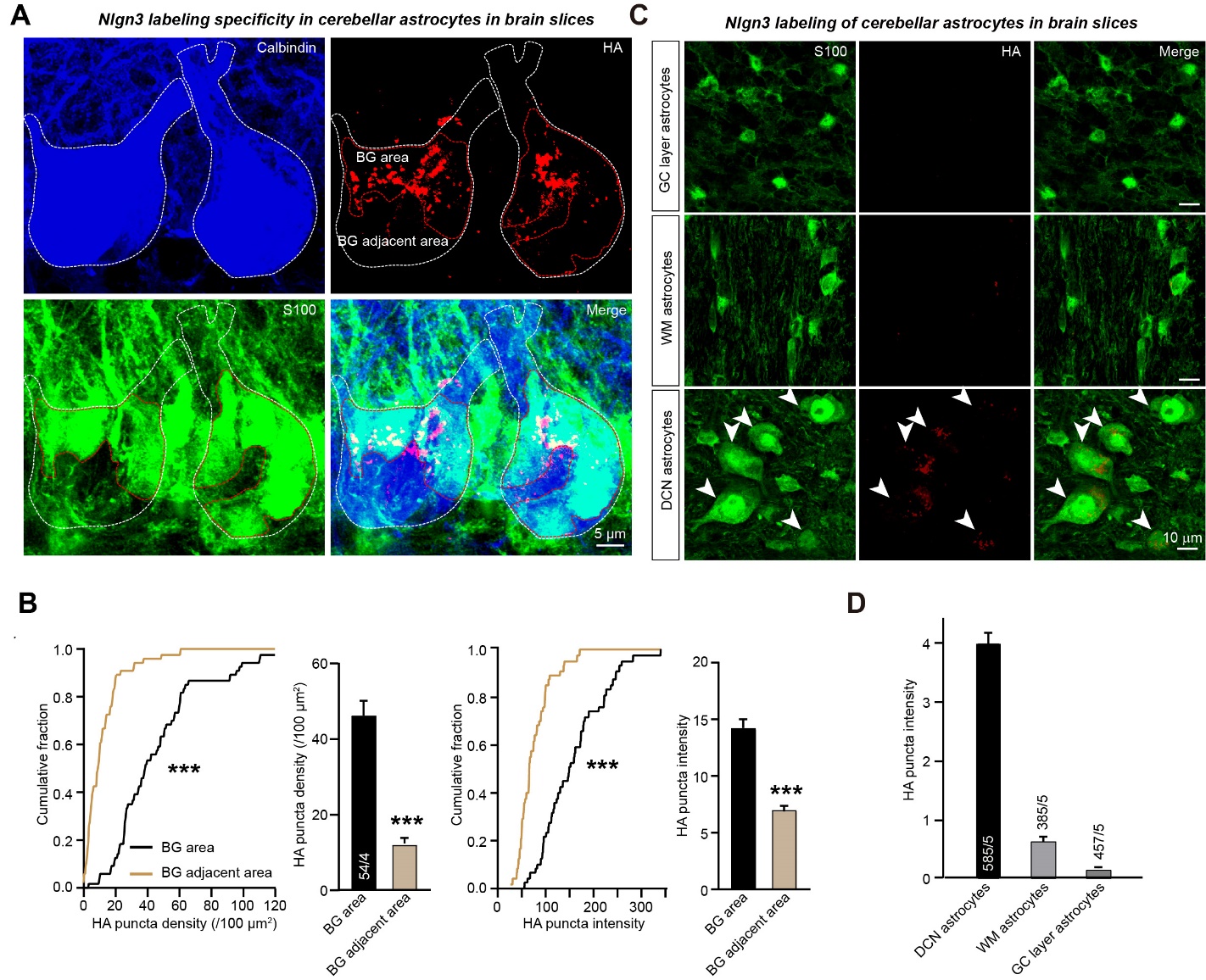


**Figure S4: Detailed analysis of Nlgn3 distribution in cerebellar astrocytes.** (**A**) Representative images showing the specificity labeling of HA in cerebellar astrocytes in brain slices. (**B**) Cumulative and summary data for the HA puncta density and puncta intensity in the BG area and BG-adjacent area in cerebellar astrocytes in brain slices from (A). (**C**) Representative images showing the co-immunostaining of HA with S100 in Granule cells (GC), white matter (WM), and deep cerebellar nucleus (DCN). Scale bar, 10 µm. (**D**) Quantitative analysis of HA co-localization ratio with S100 from (C). DCN, 585 cells from 5 mice (585/5); WM, 385 cells from 5 mice (385/5); GC, 450 cells from 5 mice (450/5). All data was presented as means ± SEM; Statistical analysis was performed by Welch's *t*-test (bar diagrams) or Kolmogorov-Smirnov test (cumulative distributions); ***P<0.001. The numbers of cells/mice (B, D) analyzed are shown in the bar graphs.


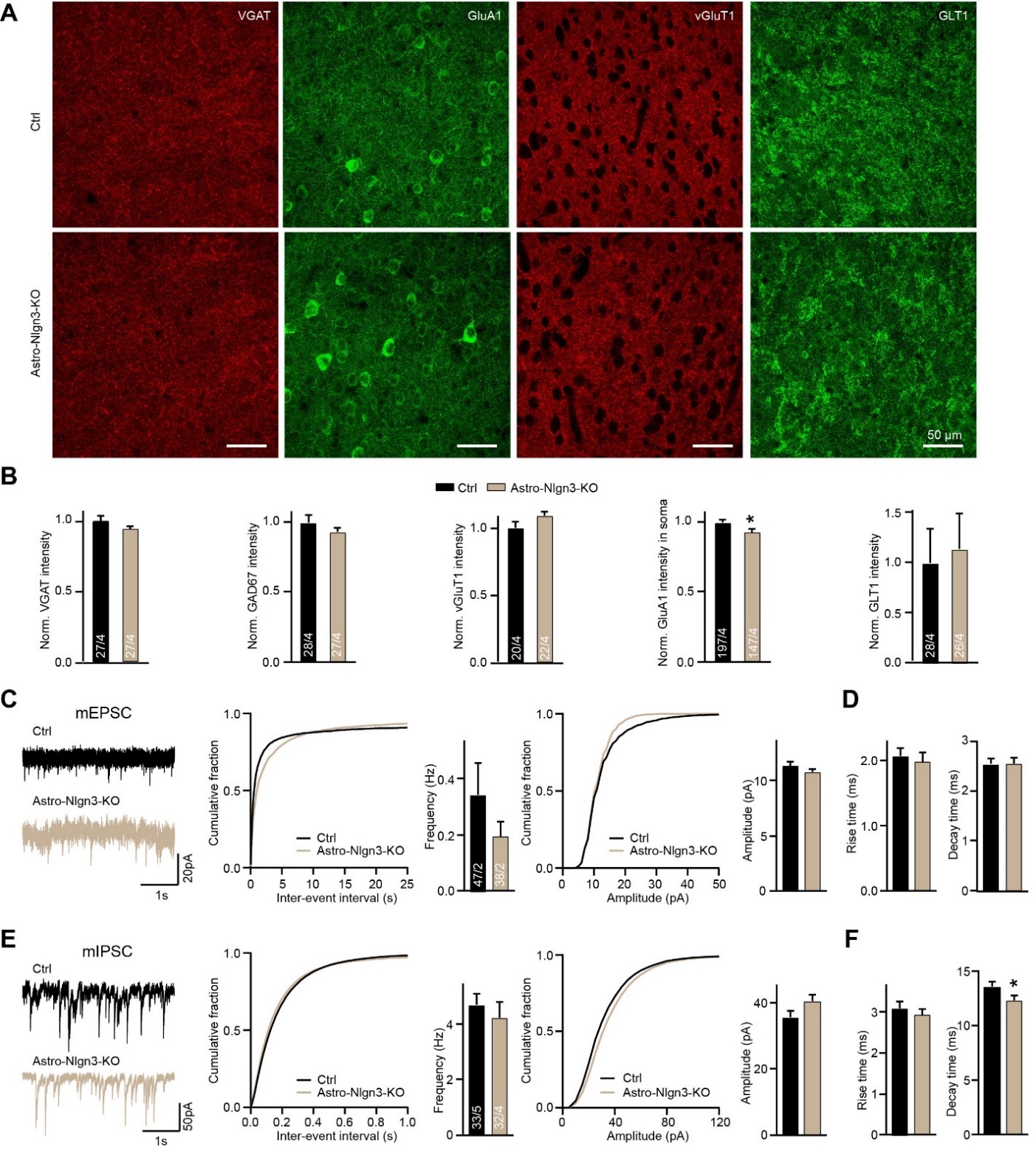


**Figure S5:** **Astrocytic Nlgn3 is dispensable for synapse numbers and synaptic transmission in the somatosensory cortex.** (**A**) Representative confocal images of synaptic markers in the somatosensory cortex from Aldh1L1-Nlgn3-KO mice and littermate control mice. Scale bars, 50 μm. Animals were injected with tamoxifen between P14 and P18 and analyzed between P24 and P28. (**B**) Summary graphs of the signals in (A). (**C-D**) Sample traces, cumulative plots, and kinetics of mEPSC recorded from P24-P28 somatosensory cortex. (**E-F**) Sample traces, cumulative plots, and kinetics of mIPSC recorded from P24-P28 somatosensory cortex. All data are shown as means ± SEM; Statistical analysis was performed by Welch's *t*-test (bar diagrams) or Kolmogorov-Smirnov test (cumulative distributions); * *p*<0.05. The numbers of sections/mice (B) or neurons/mice (C-D, E-F) analyzed are shown in the bar graphs.


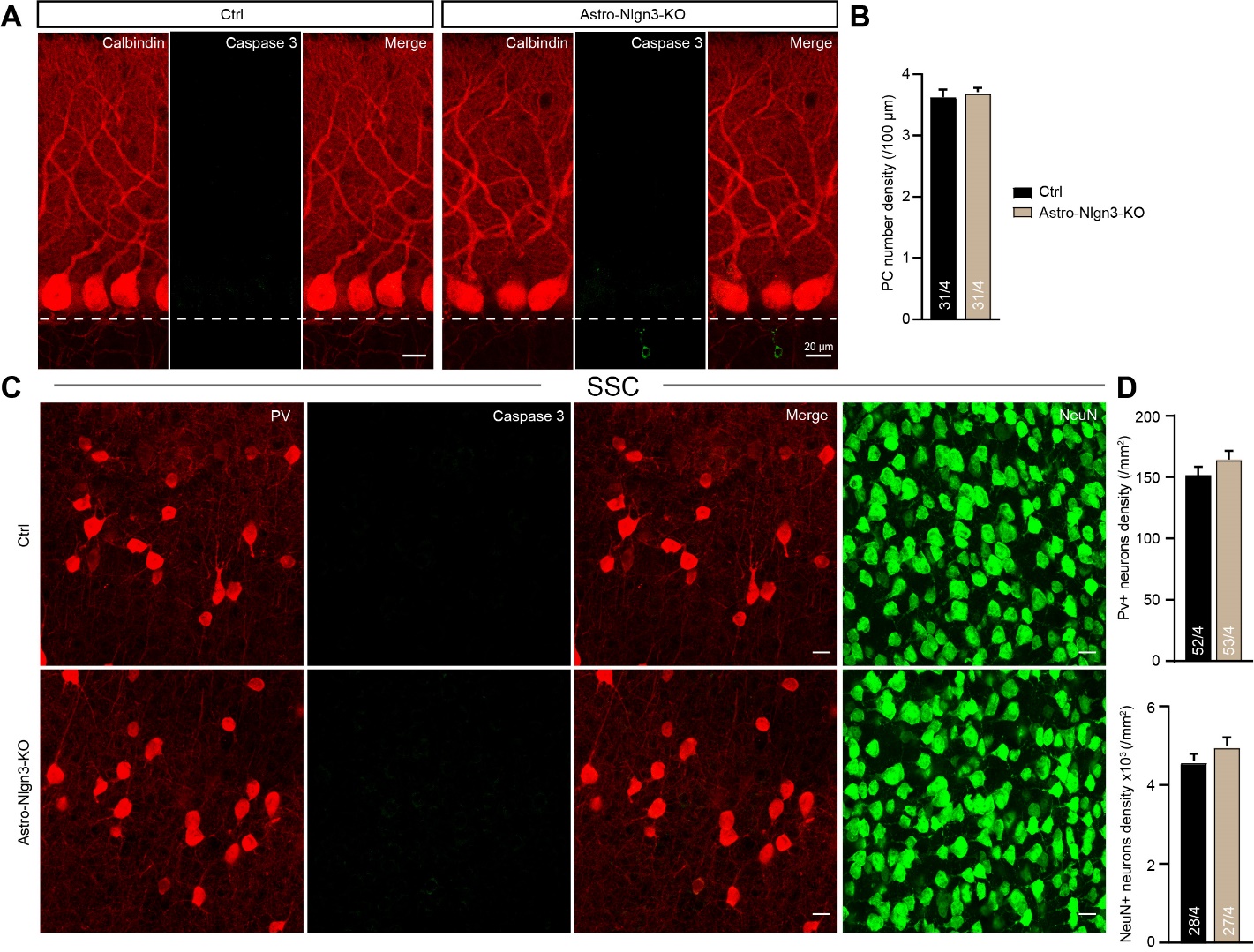


**Figure S6: The intact cell density after the deletion of Astrocytic Nlgn3 leave.** (**A**) Representative images showing immunostaining of Calbindin (red) with Caspase3 (green) in the cerebellar cortex of control and Aldh1L1-Nlgn3-KO mice. Scale bars, 20 μm. (**B**) Quantification of the density of Calbindin-positive neurons in (A). (**C**) Representative images showing immunostaining of PV (red) with Caspase3 (green) and NeuN in the somatosensory cortex of control and Aldh1L1-Nlgn3-KO mice. Scale bar, 20 μm. (**D**) Quantification of the density of PV-positive neurons and NeuN-positive neurons in (C). All data are shown as means ± SEM. The number of sections/mice analyzed is shown in the bar graphs by Welch's *t*-test.


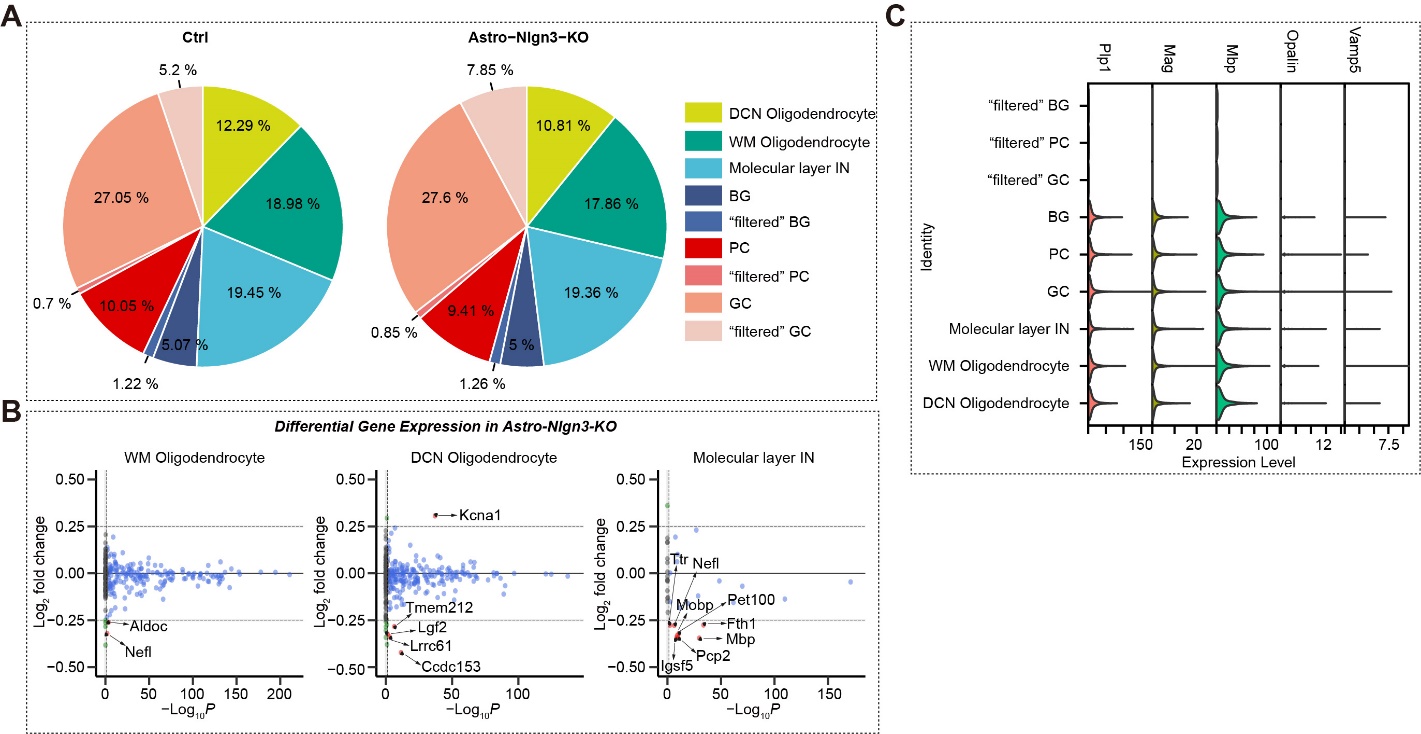


**Figure S7: Astrocytic Nlgn3 broadly regulates transcription in the cerebellum.** (**A**) Percentage of spot number of each type in Astro-Nlgn3-KO and Ctrl groups after unsupervised clustering and manual classification based on prior knowledge. (**B**) Volcano plots showed significant changes in genes caused by astrocytic Nlgn3 knockout with those unsupervised datasets in the cerebellar cortex, including WM Oligodendrocyte, DCN Oligodendrocyte, and Molecular layer IN. (**C**) Violin plots showed the expression level of *Plp1*, *Mag*, *Mbp*, *Opalin,* and *Vamp5* genes in each cell type.


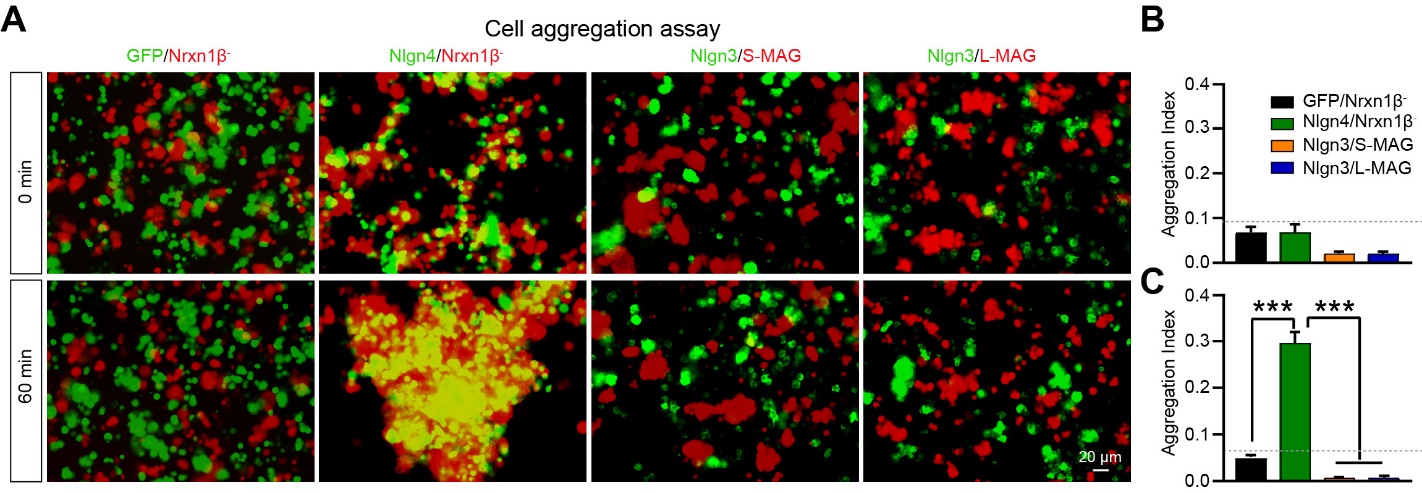


**Figure S8: Nlgn3 doesn't directly bind to S-MAG and L-MAG.** (**A**) Representative images of 0 minutes and 60 minutes incubation of GFP with Nrxn1β^-^ (negative control), Nlgn4 with Nrxn1β^-^ (positive control), Nlgn3 with S-MAG, and Nlgn3 with L-MAG. Aggregation was observed after 60 minutes in Nlgn4 with Nrxn1β^-^. (**B-C**) Quantification of the aggregation index in all conditions at 0 minutes (**B**) and 60 minutes (**C**). Aggregation index = overlap area / (area of channel 1 + area of channel 2 - overlap area) x 100. All data presented as means ± SEM; ***P<0.001 by Welch's *t*-test. The quantitative data were from 20-27 regions of interest in 4 independent cultures for all conditions.


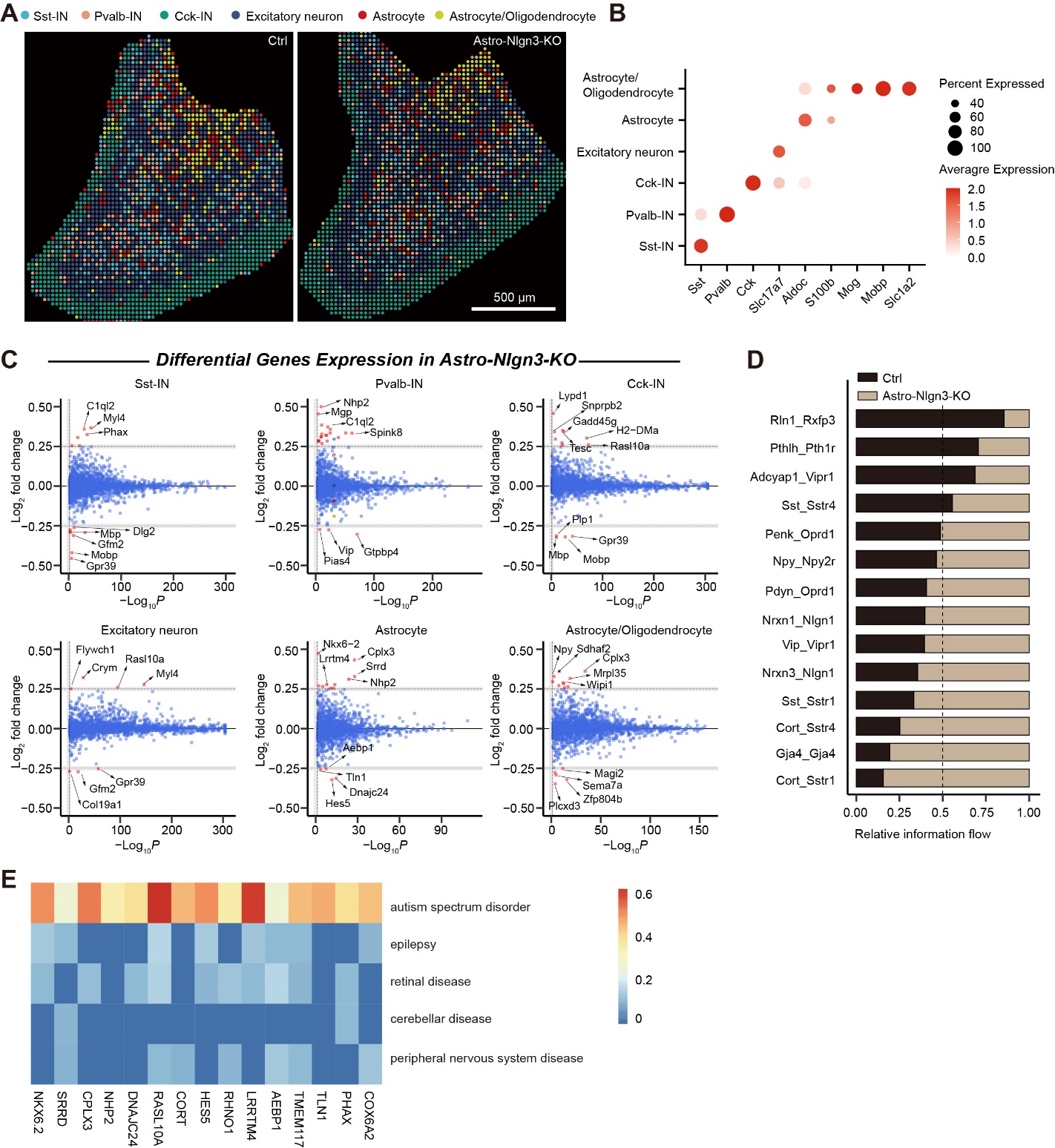


**Figure S9: Astrocytic Nlgn3 regulates transcription in the V1 cortex.** (**A**) The spatial visualization of major cell types in the V1 cortex. (**B**) Bubble plot showing the expression level of specific markers defining major cell types in the V1 cortex. Heatmap indicates the expression levels of indicated genes. The color indicates the Z score scaled gene expression levels. (**C**) Volcano plots showed significant changes in genes caused by astrocytic Nlgn3 knockout with those unsupervised datasets in the V1 cortex. (**D**) Normalized expression strength of cell communication signaling pathways among 6 cell types. (**E**) Heatmap showing the top 5 terms enriched for the 15 differentially expressed genes of cortical astrocytes. Heatmap indicates the expression levels of indicated genes. The color indicates the Z score scaled gene expression levels.

References:

1. Harris M, Jensen E. Three-dimensional structure of dendritic spines and synapses in rat hippocampus (CA1) at postnatal day 15 and adult ages. Journal of Neuroscience. 1992;12:2665–2705.

2. Zagrebelsky M, Holz A, Dechant G, Barde YA, Bonhoeffer T, Korte M. The p75 neurotrophin receptor negatively modulates dendrite complexity and spine density in hippocampal neurons. Journal of Neuroscience. 2005;25:9989–9999.

3. Restrepo S, Schwartz SL, Kennedy MJ, Aoto J. Measuring transcellular interactions through protein aggregation in a heterologous cell system. Journal of Visualized Experiments. 2020;2020:1–7.

4. Zhang B, Südhof TC. Neuroligins Are Selectively Essential for NMDAR Signaling in Cerebellar Stellate Interneurons. J Neurosci. 2016;36:9070–9083.

5. Zhang B, Chen LY, Liu X, Maxeiner S, Lee S-J, Gokce O, et al. Neuroligins Sculpt Cerebellar Purkinje-Cell Circuits by Differential Control of Distinct Classes of Synapses. Neuron. 2015;87:781–796.

6. Hashimoto K, Kano M. Postnatal development and synapse elimination of climbing fiber to Purkinje cell projection in the cerebellum. Neurosci Res. 2005;53:221–228.

7. Yamasaki M, Hashimoto K, Kano M. Miniature synaptic events elicited by presynaptic Ca2+ rise are selectively suppressed by cannabinoid receptor activation in cerebellar Purkinje cells. Journal of Neuroscience. 2006;26:86–95.

8. Hashimoto K, Ichikawa R, Kitamura K, Watanabe M, Kano M. Translocation of a ‘winner’ climbing fiber to the Purkinje cell dendrite and subsequent elimination of ‘losers’ from the soma in developing cerebellum. Neuron. 2009;63:106–118.

9. Maejima T, Hashimoto K, Yoshida T, Aiba A, Kano M. Presynaptic inhibition caused by retrograde signal from metabotropic glutamate to cannabinoid receptors. Neuron. 2001;31:463–475.
